# SUDA: A SUrface Dust Analyser for Compositional Mapping of the Galilean Moon Europa

**DOI:** 10.1007/s11214-025-01134-0

**Published:** 2025-01-29

**Authors:** Sascha Kempf, Scott Tucker, Nicolas Altobelli, Christelle Briois, Morgan L. Cable, Eberhard Grün, Murthy S. Gudipati, Bryana L. Henderson, Hsian-Wen Hsu, Kevin Hand, Mihaly Horanyi, Frank Postberg, Jürgen Schmidt, Ralf Srama, Zoltan Sternovsky, Gabriel Tobie, Mikhail Y. Zolotov, Chris Belting, Susan Bortfeldt, Jordy Bouwman, Nat Brennan, Karen Bryant, Timothy Cassidy, David Crotser, Alexandra Curtin, Elz DeVito, Donrich Ebuen, Nat Faber, Melanie Fisher, John Fontanese, Maxwell Fowle, Wendy Frank, Scott Gurst, Sally Haselschwardt, Vaughn Hoxie, Karl Hubbell, David James, Mark Kien, Scott Knappmiller, Rick Kohnert, Alexander Lampe, Mark Lankton, Sean Lev-Tov, Crystal McGinn, Marc Miller, Gregory Newcomb, Samuel Oberg, Leela O’Brien, Kathrine Pilewskie, Shawn Polson, Victoria Scarffe-Barrett, David Summers, Stacy Wade, Alexandria Ware, Alan Yehle, Corinne Wuerthner, Adrian Garcia Arteaga, Bogdan Oaida, Chad Eberl, Polly Fitton, William Goode, Zuni Levin, Gwyneth Lowry, Jared Stanley, Anthony Tracy, Zach Ulibarri, Ethan Williams, Camille Yoke, Ben S. Southworth, Jonathan K. Hillier, Nozair Khawaja, Fabian Klenner, Maryse Napoleoni, Jonas Simolka, Jason Sioeng

**Affiliations:** 1 LASP, University of Colorado, 1234 Innovation Drive, Boulder, CO 80303 USA; 2 Shreve Research, Boulder, CO USA; 3 ESAC, Camino bajo del Castillo, Villanueva de la Cañada, Madrid, Spain; 4 UMR-CNRS 7328, LPC2E, OSUC, 45071 Cedex 2 Orleans, France; 5 Jet Propulsion Laboratory, California Institute of Technology, 4800 Oak Grove Drive, Pasadena, CA 91109 USA; 6 Institut für Geologische Wissenschaften, Freie Universität Berlin, Malteserstrasse 74-100, Berlin, 12249 Germany; 7 Division for Astronomy, Department of Physics, University of Oulu, Oulu, Finland; 8 Department of Earth and Space Sciences, University of Washington, 4000 15th Avenue NE, Seattle, WA 98195 USA; 9 IRS, Universität Stuttgart, Pfaffenwaldring 31, D-70569 Stuttgart, Germany; 10 Theoretical Division, Los Alamos National Laboratory, Los Alamos, USA; 11 LPG, UMR 6112, CNRS, Nantes University, Nantes, France; 12 School of Earth and Space Exploration, Arizona State University, Tempe, 85287-1404 AZ USA; 13 California State Polytechnic University, Pomona, 3801 W Temple Ave, Pomona, CA 91768 USA

## Abstract

The Surface Dust Analyser (SUDA) is a mass spectrometer onboard the Europa Clipper mission for investigating the surface composition of the Galilean moon Europa. Atmosphereless planetary moons such as the Galilean satellites are wrapped into a ballistic dust exosphere populated by tiny samples from the moon’s surface produced by impacts of fast micrometeoroids. SUDA will measure the composition of such surface ejecta during close flybys of Europa to obtain key chemical signatures for revealing the satellite’s composition such as organic molecules and salts, history, and geological evolution. Because of their ballistic orbits, detected ejecta can be traced back to the surface with a spatial resolution roughly equal to the instantaneous altitude of the spacecraft. SUDA is a Time-Of-Flight (TOF), reflectron-type impact mass spectrometer, optimized for a high mass resolution which only weakly depends on the impact location. The instrument will measure the mass, speed, charge, elemental, molecular, and isotopic composition of impacting grains. The instrument’s small size of $268 ~\mathrm {mm} \times 250 ~\mathrm {mm} \times 171$$~\mathrm {mm}$, radiation-hard design, and rather large sensitive area of 220 cm^2^ matches well the challenging demands of the Clipper mission.

## Introduction

The SUrface Dust Analyzer (SUDA) was selected in 2015 for the instrument payload of NASA’s Europa Clipper mission to the Galilean moon Europa (Pappalardo et al. 2024, this collection). The giant ice moon is believed to harbor a subsurface liquid water reservoir between its ice crust and silicate core (Carr et al. 1998; Khurana et al. 1998; McCord et al. 1998). Because liquid water constitutes an essential prerequisite for the emergence of life, the Europa Clipper mission will explore the geologically active moon in depth. The overarching mission goal is to assess Europa’s habitability, and SUDA will contribute to the mission success through the completion of three Level-2 requirements, which are summarized in Table 1 and correspond to the Level-1 requirements listed therein. The lower-level science measurement requirements that map to the Level-2 requirements are provided in Appendix A.

**Table 1:** SUDA-relevant Level-1 requirements and summaries of Level-2 science requirements for the Europa Clipper mission

Theme	Level-1 baseline requirements	Level-2 science requirements
Global Composition	Create a compositional map at $\le 10 ~\mathrm {km} $ spatial scale cover ≥60% of the surface, sufficient to identify non-ice materials, especially organic compounds.	Map compositionally diagnostic properties in the in situ volatile and (ice/dust) particle datasets to determine the surface composition and chemistry, including the identification of any hydrated minerals and organic compounds, and seek indicators of ocean geochemical processes relevant to habitability.
Atmospheric Composition	Characterize the composition and sources of volatiles, particulates, and plasma, sufficient to identify the signatures of non-ice materials, including organic compounds, in at least one of the above forms, in globally distributed regions of the atmosphere and local space environment.	Characterize the composition of near surface exospheric ice/dust particles, including any organic compounds if present, and to distinguish between exogenic and endogenic sources of material across globally-distributed regions and in unique geographical locations.
Current Activity	Search for and characterize any current activity, notably plumes or thermal anomalies, in regions that are globally distributed.	Identify and characterize potential recent and/or ongoing activity in any encountered plumes $<110 ~\mathrm {km} $ in altitude; determine composition including organic compounds if present, number density, and size distribution of any plume ice/dust particles to identify and constrain the plume’s source mechanism and salinity of the source.

SUDA is a high resolution, dual polarity Time-Of-Flight (TOF) impact mass spectrometer, derived from the heritage of dust instruments on Cassini (Srama et al. 2004), Giotto (Kissel 1986), Rosetta (Kissel et al. 2007), and Stardust (Kissel et al. 2004). SUDA will measure the mass, speed, charge, elemental, and isotopic compositions of impacting grains originating from Europa’s surface. The engineering model as well as the flight instrument have been designed, fabricated, and qualified by a team at the Laboratory for Atmospheric and Space Physics (LASP) at the University of Colorado at Boulder.

This paper provides a summary of the scientific background and SUDA measurement requirements in Sect. 2 and the engineering details of the instrument in Sect. 3. The results of the instrument performance presented in detail in Sect. 4. These tests demonstrate that SUDA is capable of delivering the required measurements. In Sect. 5, we provide further details about the instrument operation, the SUDA data products, and the implementation of the SUDA science acquisition.

## Science Background

The composition of dust particles provides unique insights into the physical and chemical conditions at their origin. For example, the Cosmic Dust Analyser (CDA) (Srama et al. 2004) onboard Cassini at Saturn detected a variety of sodium salts from embedded minerals in the ice grains entrained in the plume of Enceladus (Postberg et al. 2009, 2011b, 2023), stemming from the moon’s rocky core, which is, or has been, in contact with a reservoir of liquid water (Zolotov 2007). This finding provides evidence that a liquid subsurface water reservoir is the source of the emitted grains, which could not have been obtained by traditional remote sensing techniques. Sodium eluded detection in the emerging gas plume, by both the Cassini Ion and Neutral Mass Spectrometer (INMS) (Waite et al. 2006, 2009) and by ground-based observations (Schneider et al. 2009) due to the fact that the majority of the sodium is present in the form of non-volatile salts within the ice grains. Therefore, in-situ measurements of gases in the exosphere around a planetary body are greatly enhanced by complementary compositional analysis of the dust particles emerging from its surface, which provides constraints on the nature of any geological activity. Similarly, remote sensing observations are insufficient for resolving these issues. To gain a detailed understanding of these processes and to evaluate the composition of a subsurface ocean, it is necessary to conduct direct sampling of the material on Europa’s surface and/or plumes, if present. Nadir-pointing imaging and spectral instruments, in combination with a ram-pointing dust detector and neutral gas mass spectrometer, observe the same real estate and provide data sets that are complementary to one another, thus enabling the unambiguous identification of the chemical constituents of surface materials.

**Fig. 1:**
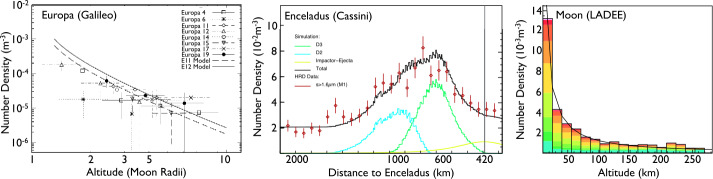
Ejecta clouds around moons without an atmosphere are a common phenomenon in the solar system. **Left:** Europa’s ejecta cloud was present during all Galileo flybys (Krüger et al. 2003a). **Middle:** Saturn’s moon Enceladus maintains an ejecta cloud beside the ice particle plume at its south pole (Kempf et al. 2010). **Right:** Earth’s Moon also maintains a pronounced ejecta cloud (Horányi et al. 2014a)

Inspired by the successes of the Cassini dust mass spectrometer, a new method for obtaining detailed information about the surface composition of airless planetary bodies has been developed (Kempf 2009; Postberg et al. 2011a; Goode et al. 2021, 2023). The compositional mapping technique relies on the fact that impacts of fast, typically 100$~\textrm {\textmu }\mathrm {m}$ interplanetary meteoroids on the moon’s surfaces produce ejecta particles which populate a tenuous, approximately spherically symmetric cloud around the moon (Krivov et al. 2003; Sremčević et al. 2003, 2005). Information about the geological activities at and below Europa’s surface, in particular the material exchange between the interior and the surface, is likely contained in the types and amounts of inorganic and organic components embedded in the surface. This method is readily available for the Europa Clipper mission, as the ejecta dust clouds have already been detected – albeit without compositional information – around all the icy moons of Jupiter by the Galileo dust detector (Krüger et al. 2003c, see Fig. 1).

The overarching Europa Clipper mission goal is to gauge Europa’s habitability (Pappalardo et al. 2024; Vance et al. 2023; both this collection). The composition of its surface and subsurface material may hold fundamental clues for understanding its potential to develop and sustain life (Becker et al. 2024, this collection). The surface of Europa is globally covered with water ice (Hall et al. 1995; Roth et al. 2017). Materials embedded in the ice matrix of Europa’s surface likely carry a treasure trove of information about the moon’s interior (Becker et al. 2024, this collection). The composition of the surface material may reflect both exogenic and endogenic processes. Exogenic processes include radiolysis, implantation of molecules and ions from the Jovian magnetosphere, and dust impacts. These exogenic processes are responsible for the formation of strong oxidants ($\mathrm {O_{2}}$, $\mathrm {H_{2}O_{2}}$) in irradiated ices as well as the accumulation of molecules and elements from Io ($\mathrm {SO_{2}}$, Na, K, Cl) contaminating Europa’s surface together with chondritic and cometary materials containing both inorganic and organic components (Carlson et al. 2009). Endogenic processes are likewise responsible for the delivery of non-ice materials, best manifested in disrupted surface features: ridges, chaotic terrains, pits, and domes. Galileo and telescopic IR data suggest the presence of hydrated sulfate species: magnesium and sodium sulfates along with sulfuric acid hydrates (McCord et al. 1998; Carlson et al. 2009; Shirley et al. 2010; Brown and Hand 2013). $\mathrm {CO_{2}}$ is reported in colored non-ice materials (McCord et al. 1998; Hansen and McCord 2008). The detection of $\mathrm {CO_{2}}$ in within the recently resurfaced Tara Regio suggests that the carbon originates from an internal source (Trumbo and Brown 2023; Villanueva et al. 2023). The detection of Na and K in the exosphere of Europa (Brown 2001) implies their origin in surface materials (Cipriani et al. 2009). Visible-wavelength spectra obtained with the Hubble Space Telescope suggests that the surface of Europa may contain $\mathrm {NaCl}$ and $\mathrm {MgSO_{4}}$ (Trumbo et al. 2020). These observations are consistent with geochemical models which predict abundant alkali, magnesium, sulfates, chloride, and carbonate species dissolved in a global subsurface European ocean (Kargel et al. 2000; Zolotov and Shock 2001, 2004; Soderlund et al. 2020; Wolfenbarger et al. 2022). SUDA will constrain the ratio of exogenic to endogenic material on Europa’s surface by measuring the exogenous mass flux onto the surface. Understanding of surface-altering processes and the evaluation of the composition of the ocean requires the direct sampling of the surface because remote sensing alone is unable to resolve these issues, as previously mentioned.

A fundamental aspect of evaluating the habitability of Europa is to ascertain the capacity of its putative ocean habitat to furnish the essential elements and chemical energy indispensable for life (Hand et al. 2009). The mere detection of organic molecules is inadequate for achieving this objective. Organic compounds are present on a multitude of objects throughout the solar system, the majority of which are not presumed to be habitable (e.g., on Phoebe, see Clark et al. 2005). Relating composition to subsurface habitability requires knowledge of both the organic and inorganic inventory in surface materials (Vance et al. 2023, this collection). SUDA is uniquely capable of providing both.

SUDA is capable of detecting a diverse range of compounds present on Europa’s surface, spanning a concentration range from percent to parts per million (ppm), and subsequently tracing their origin on the surface. This enables the simultaneous compositional mapping of numerous organic and inorganic components, including both major and trace compounds, with a single instrument. Any recent tectonic activity, cryovolcanism, or resurfacing event (e.g., a plume deposit) is detectable by variations in the surface composition over multiple flybys. This can be linked to corresponding geological features, including the analysis of compositional variations across large craters on Europa. SUDA will further the understanding of how Europa’s surface couples to its interior source regions. SUDA will analyze the surface material with a spatial resolution of less than the instantaneous spacecraft altitude ($\ge 25 ~\mathrm {km} $) and associate its composition with geological structures, albedo features, and other distinct surface areas. SUDA’s velocity sensor (Sect. 3.2.5) will distinguish exogenous dust particles from dust originating from Europa based on the order of magnitude difference in velocity.

The discovery that Europa maintains a possibly intermittent water plume (Roth et al. 2014) offers an additional unique scientific opportunity to understand the conditions at Europa’s subsurface ocean (or other near-surface reservoir). It is noteworthy that the water column densities of the plumes from Enceladus (Hansen et al. 2011) and Europa are comparable, suggesting that Europa’s plume may also contain a few mass percent of water ice particles. These particles are formed through two primary mechanisms: (i) nucleation within the vapor streaming through fractures in Europa’s ice crust (Schmidt et al. 2008) and (ii) mantle growth on shock-frozen oceanic droplets released from the interface of the moon’s subsurface ocean (Postberg et al. 2011b). An ice particle plume of this kind would have a scale height of approximately 50$~\mathrm {km}$ (Southworth et al. 2015), enabling the Europa Clipper in-situ detectors to collect samples during traversals of the plume by the spacecraft. SUDA is uniquely capable of determining the composition of the frozen droplets originating from subsurface water reservoirs from collected ice particles.

### Composition of Surface Ejecta

**Fig. 2:**
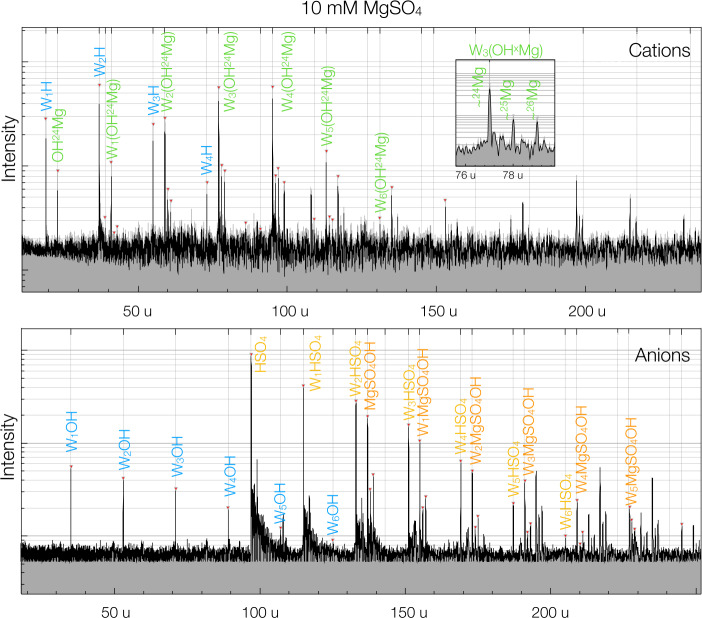
Laser-assisted dispersion spectra of 10 mM $\mathrm {MgSO_{4}}$ in water (Napoleoni et al. 2023), which are analogous to impact mass spectra (Postberg et al. 2009). $\mathrm {MgSO_{4}}$ is a hydrated mineral likely to be found on Europa’s surface. In addition to the dominant $\mathrm {(H_{2}O)_{n}H^{+}}$ ion clusters, the cation spectrum also shows clusters formed from the metallic component Mg of $\mathrm {MgSO_{4}}$ and water. The inset shows the three main isotopologues of $\mathrm {(H_{2}O)(MgSO_{4})}$ appearing in the cation spectrum. The anion spectrum shows water cluster ions of $\mathrm {SO_{4}^{-}}$. Note that despite the low concentration of $\mathrm {MgSO_{4}}$ in the ice matrix, it appears in corresponding cluster ions at intensities $> 1\%$ in the mass spectra. This fact enables unambiguous detection of ppm-level or ppb-level concentrations of salts and polar organic compounds with impact mass spectrometers

The vast majority of the dust grains launched by the impact-ejecta mechanism are comprised of surface material with little or no alteration caused by the impact itself (Koschny and Grün 2001). From ejection at the surface until detection by SUDA, the molecular structure of the sample stays intact, allowing the investigation of surface composition in situ and in its original state. Even complex inorganic and organic molecules, such as amino acids or polystyrene, survive both the impact process which releases them and SUDA detection at typical speeds ($4 \ldots 5 ~\mathrm {km\,s^{-1}} $) (Srama et al. 2009b) and can be identified in SUDA’s TOF mass spectra (Fig. 2). These spectra show the molecular composition of individual surface samples over a mass range from 1 to 500$~\mathrm {u}$ in both positive (cation) and negative (anion) channels. In contrast to the elemental makeup, the molecular composition makes it much more straightforward to determine the true chemical structure of surface material and distinguish exogenic from endogenic materials (both inorganic and organic). Note also that exogenic dust particles arrive at a much larger speeds than ejecta particles, providing another indicator with which to constrain their origin.

Currently, the composition and amounts of non-ice materials exposed at Europa’s surface are only vaguely constrained. Available information about non-ice surface materials mainly stems from the interpretation of IR surface spectra, which is prone to uncertainties. In particular, no set of compounds studied so far in the laboratory has satisfactorily matched any of the Galileo IR observational data (Dalton et al. 2010). The interpretation of the TOF impact mass spectra of ejected surface materials is far less ambiguous, as the molecular composition can be identified by a characteristic pattern of mass lines. In addition to identification, the quantitative analysis of surface composition is the key objective of SUDA, and one that is not possible with remote sensing techniques alone. Inorganic compounds embedded in the surface’s ice matrix such as sulfuric acid, hydrated sulfates and other salts, such as alkali chlorides, are most likely to appear in the SUDA spectra as water cluster cations (Fig. 2) (Napoleoni et al. 2023), similar to what is observed with sodium salts in Saturnian ice particle spectra (Postberg et al. 2009, 2023).

**Fig. 3:**
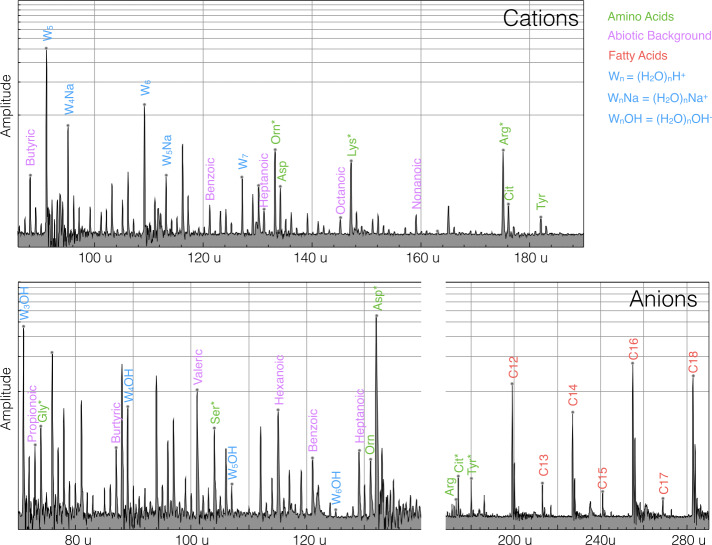
Laser-assisted dispersion spectra of salty water containing amino acids (Asp, Glu, His, Arg, Cit, Gly, Ser, Thr, Orn, and Tyr) and fatty acids (C12 … C18) at relative abundances chosen to be representative for biotic processes. The mixture is considered to be representative for Enceladus ice grains formed by shock-freezing bubbles at the boiling subsurface ocean interface. The concentrations of the amino acids is $\sim 1 $ ppmw. The data are from Klenner et al. (2020a). More recent experiments show similar results using cell material extracted from the Escherichia coli model organism and the psychrophile Sphyngopyxis alaskensis (Dannenmann et al. 2023)

Although the presence of organics at Europa’s surfaces is likely, it is currently unclear which specific compounds are to be expected. The Cassini CDA results for Enceladus revealed the presence of large organic molecules (Postberg et al. 2018) as well as smaller reactive organic molecules (Khawaja et al. 2019) in the ice grains emerging from the Enceladus plume. Laboratory experiments show that even trace amounts of complex organic molecules in the ice matrix can be identified in the mass spectra (Klenner et al. 2020a,b; Ulibarri et al. 2023) (Fig. 3). In recent experiments even single-celled organisms embedded in grains led to signatures in mass spectra known to be characteristic for biologic activity, such as the odd-even pattern of fatty acids from cell membranes (Dannenmann et al. 2023).

### Mass Flux of Refractory Material onto Europa

Knowledge about the mass flux of exogenous material on Europa’s surface is essential for associating Europa’s surface composition with endogenic processes. SUDA is tasked with measuring the flux of material originating from Io and the minor moons as well as interplanetary and interstellar micrometeoroids. Solid material from Io’s volcanoes propagates through the Jovian system as so-called stream particles that escape into interplanetary space. These have been discovered by Ulysses as high-speed streams of nano-grains (Grün et al. 1993). Subsequent long-term monitoring of the dust stream from within the Jovian magnetosphere by Galileo (1996-2003) led to the recognition that charged nano-grains mostly originate from Io’s volcanoes (Graps et al. 2000). The compositional data obtained by CDA during the Cassini flyby of Jupiter in 2000 demonstrated that most of the Io grains are predominantly composed of NaCl, believed to be high-temperature condensates forming in Io’s large volcanic plumes (Postberg et al. 2006).

The charging and subsequent dynamics of small dust particles are dictated by the electromagnetic environment of Jupiter, effectively turning them into swarms of plasma probes that will be analyzed by SUDA. The long-term temporal and spatial variability of the dust fluxes are shaped by the orbital motion of their source, Io, and the longitudinal asymmetry of the Jovian plasma torus (Horányi et al. 1997; Krüger et al. 2003b). In contrast, the short-term, large deviations from the secular behavior of the stream dust particle fluxes are related to the stochastic nature of Io’s volcanic activity (Krüger et al. 2003b). SUDA will identify periods of enhanced volcanic activity on Io and measure the flux and composition of Io particles (see Sect. 5.3.3). Moreover, SUDA will ascertain whether there are additional sources of nano-grains, such as the moons Thebe and Amalthea, or the Gossamer rings (Hamilton and Burns 1993), and determine their composition.

Another important source of exogenous material are ejecta particles from the Galilean moons Callisto and Ganymede that have escaped into the so-called Galilean ring (Krivov et al. 2002) and collided with Europa. The tenuous ring extends inside Europa’s orbit to beyond Callisto’s orbit. Liu et al. (2016) have numerically studied the transport of dust between the Galilean moons and determined the spatial distribution of the ring particles as a function of their origin. Because SUDA can identify the source body of a detected ring particle on the basis of its composition, the predictions by Liu et al. (2016) can be verified and the mass flux of Ganymede and Callisto material onto Europa will be constrained by SUDA’s observations (see Sect. 5.3.2).

### Surface Composition Mapping

During Europa flybys, SUDA will collect compositional data from Europa’s ejecta cloud that can be directly associated with their source locations on the moon’s surface. To relate composition to geology, we will build a compositional map for geological features located around closest approach along the ground-track of the flyby. This is especially useful for establishing ground truth for young features that likely bear compositional characteristics that represent young subsurface material (Wilson et al. 1997; Mével and Mercier 2007). We have developed tools to map series of SUDA detections of different compositional types to geological features to provide robust compositional abundance estimates within a given feature (Goode et al. 2023). These abundance measurements are spatially resolved for features (or designated Unique Geological Locations (UGLs) along the ground-track of a low-altitude flyby (< 35 km) near closest approach.

**Fig. 4:**
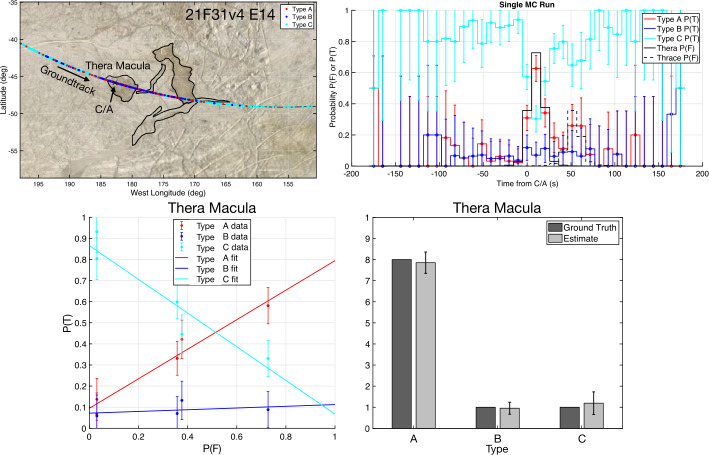
Monte-Carlo simulation of SUDA measurements during Europa Clipper flyby E14 with a 35$~\mathrm {km}$ closest approach (Table 10) (Goode et al. 2023). The ground track (top left panel) passes through the approximate center of Thera Macula with a closest approach (C/A) inside the feature. As an example, it is assumed here that SUDA collects 3 different types of materials from inside and outside Thera Macula. Type A is the dominant type in the feature while there are also small amounts of types B and C. The surrounding terrain’s composition is dominated by type C while including small amounts of types A and B. SUDA collects a time series of the three types. Detections arrive at random time intervals while having random sites of origin on the surface. The task at hand is to decipher the mixed time series of types in order to determine the most likely spatial distribution of types on the surface. Goode et al. (2021, 2023) apply a Monte Carlo (MC) method to derive the probability of detecting a composition type, $P(T)$, based on a realistic time series (top right). The probability of a detection originating from a feature $P(F)$ is also shown. The relation (bottom left) between $P(T)$ (derived from SUDA data) and $P(F)$ (derived from simulation) will be used to estimate the abundance of each detected compositional type within a feature such as Thera Macula (bottom right)

Most SUDA detections originate from within the nadir-projected circle on the surface with a radius at least equal to the instantaneous altitude of the spacecraft (Postberg et al. 2011a; Goode et al. 2021). As long as the radius of this circle is comparable to that of a geological feature at or near closest approach, SUDA’s data can be spatially applied to compositional mapping (Fig. 4) and subsequently combined with remote sensing maps. The probability of a series of detections originating exclusively from a feature is derived using Monte Carlo simulations of ejecta detections on a given flyby. To ensure SUDA’s measurement requirements are met (namely, SUD.014, SUD.018, and SUD.019 from Table 9) in Europa Clipper’s tour design, our simulations of SUDA detections are used to model the ability to map the composition of unique geological features identified as high priority targets (e.g., Thera Macula shown in Fig. 4).

## Instrument Description

### Overview:

SUDA is a TOF impact mass spectrometer (Fig. 6) derived from previously flown dust compositional analyzers on Giotto, Stardust, and Cassini (Kissel et al. 2003; Srama et al. 2004). SUDA builds upon the technology of the Cosmic Dust Analyzer (CDA), which was successfully operated on Cassini, but employs advanced reflectron-type ion optics to achieve enhanced mass resolution. The instrument performance parameters are driven by the SUDA Level-2 measurement requirements given in Appendix A.

**Fig. 5:**
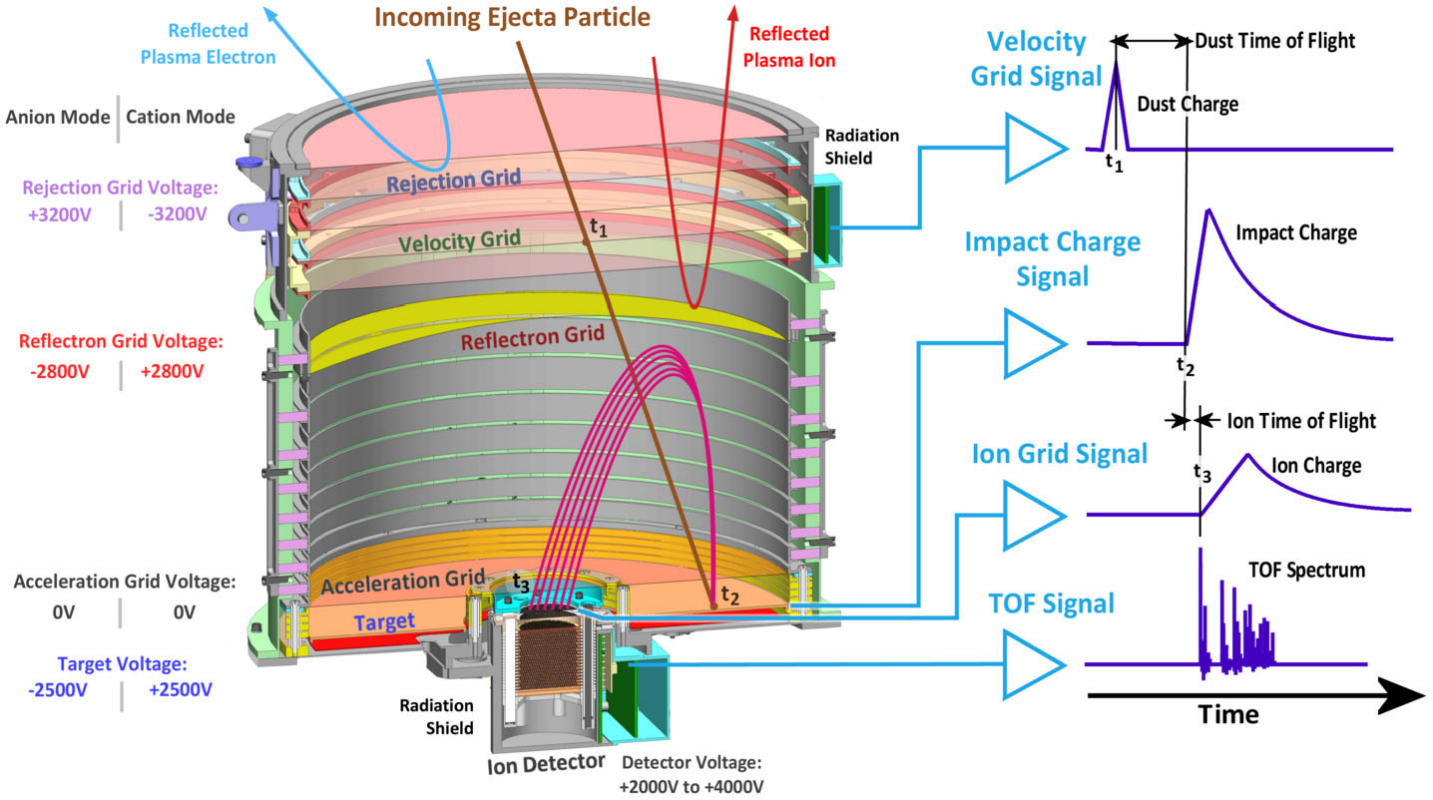
Cut-away view of SUDA illustrating the operation principle. The charge pick-up signal from one of the grid electrodes is used for velocity measurement of the incoming dust particle. The impact-generated ions are accelerated by the applied bias voltage and focused by the electrostatic field onto the detector for TOF measurements. The timing and combination of signals are used for coincident impact detection and yield the boresight component of the dust velocity, mass (determined from the impact charge) and the TOF mass spectrum. The positive/negative ion spectrum is measured by reversing the polarities of the applied bias voltages

The operation principle is illustrated in Fig. 5. Individual dust particles enter the instrument from the top, pass through a set of grid electrodes, and impact the target at the bottom. Applied bias voltages on discrete grid electrodes repel ambient plasma particles with a kinetic energy $<3 ~\mathrm {keV} $ from entering the instrument. The velocity grid, which is electrically isolated and connected to a Charge Sensitive Amplifier (CSA), provides the start signal for the TOF velocity measurement of incoming particles. CDA on Cassini has successfully measured dust velocities with comparable electrode configurations (Auer et al. 2002; Kempf et al. 2004, 2006).

**Fig. 6:**
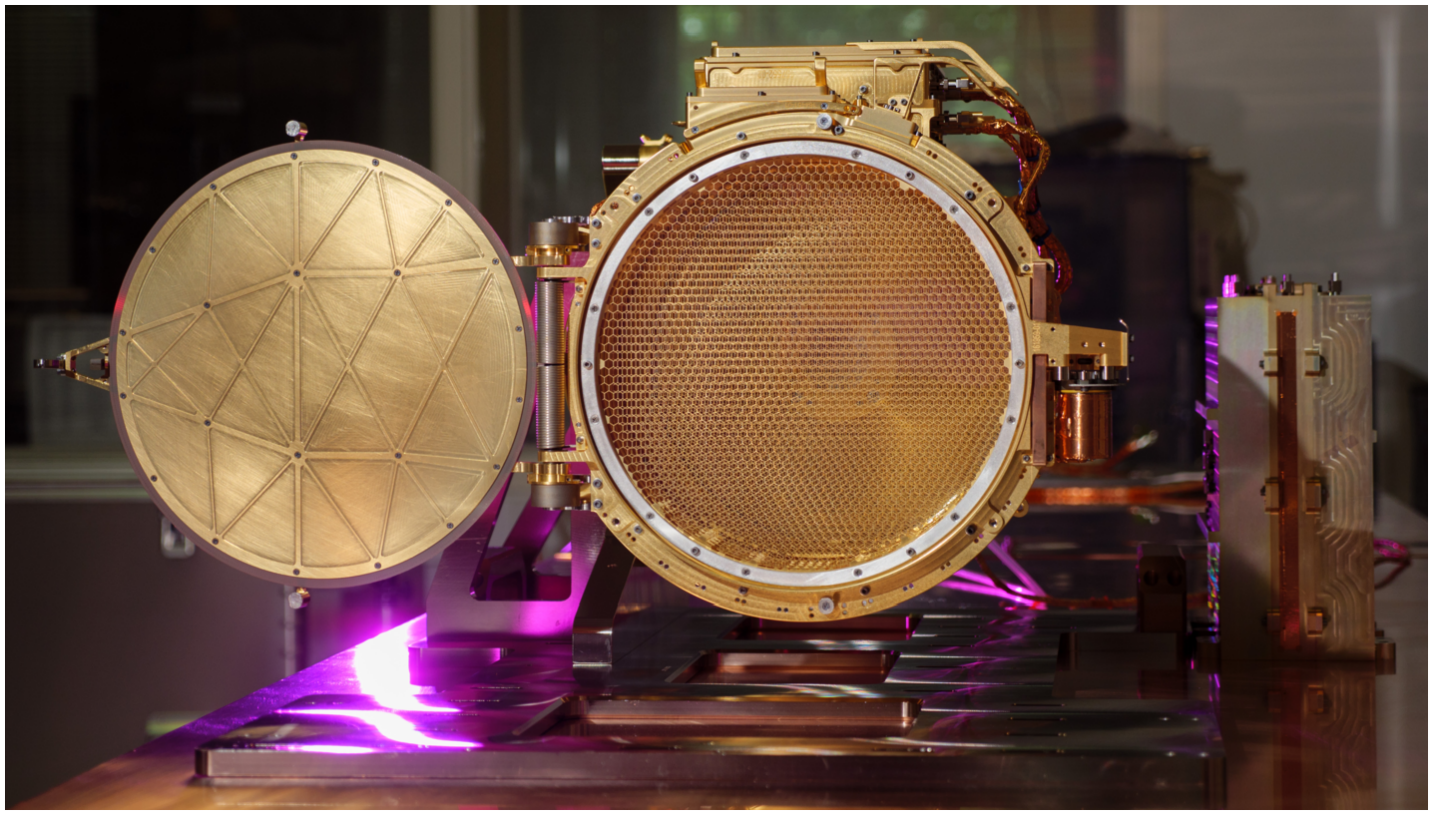
SUDA Flight Model

The impact-generated ions, which represent the dust particle composition, are accelerated by an electric field from the $+2.5 ~\mathrm {kV} $ bias potential ($-2.5 ~\mathrm {kV} $ in negative ion mode) applied to the target and the grounded acceleration grid. The impact-generated electrons are collected on the target, or show up at the beginning of the TOF signal, contingent on the polarity applied. A reflectron-like electric field (Mamyrin et al. 1973) is generated by a set of biased ring electrodes and a paraboloid-shaped grid electrode on top, which provide spatial and temporal focusing. A centrally placed ion detector collects the ions in a TOF fashion. The ion optics has been optimized for the combination of high mass resolution, large target area, and large FOV. Numerical simulations were employed to identify the optimal bias voltage of 2680$~\mathrm {kV}$ for the parabolic grid (Kempf et al. 2012a). The instrument design is robust, and minor variations in the bias potentials and/or geometric imperfections have a negligible impact on the mass resolution (Sect. 3.2.2).

#### Impact Detection and Signals Measured

SUDA employs the coincident detection method – proven successful on CDA – for unambiguous recognition of valid dust impacts. The method entails the continual monitoring of all analog signals with threshold detection. The acquisition of data is triggered by programmable threshold levels. The recorded signals are further validated by a configurable set of rules, such as a combination of threshold detections or the identification of mass lines in the TOF spectrum. The threshold levels and validation rules are determined from the environmental testing of the instrument and adjusted throughout the mission, allowing for optimal operation in different environments. In the order of events (see Fig. 5), the measured signals are as follows:

*Velocity grid signal*: The induced image charge of the incoming dust particle is detected on the velocity grid by a CSA. The relative timing of this signal yields the precision measurement of the perpendicular component of the velocity vector (see Sect. 4.2).
*Impact charge signal*: The impact charge generated at the target is collected by a CSA. The amplitude of this signal is a function of the mass and speed of the particle (e.g., Auer 2001) (see Sect. 4.3).
*Ion grid signal*: The fraction of ions focused onto the ion detector is measured on a grid electrode using a CSA. This is a low noise ion signal due to the small size and low capacitance of the collecting electrode (Kempf et al. 2005) (see Sect. 4.3).
*TOF signal*: The TOF ion signal is measured at the ion detector anode. A gain stage amplifier provides three parallel signals for a wide dynamic range with different gains (see Sect. 4.1.2).

### Mechanical and Thermal Design

**Fig. 7:**
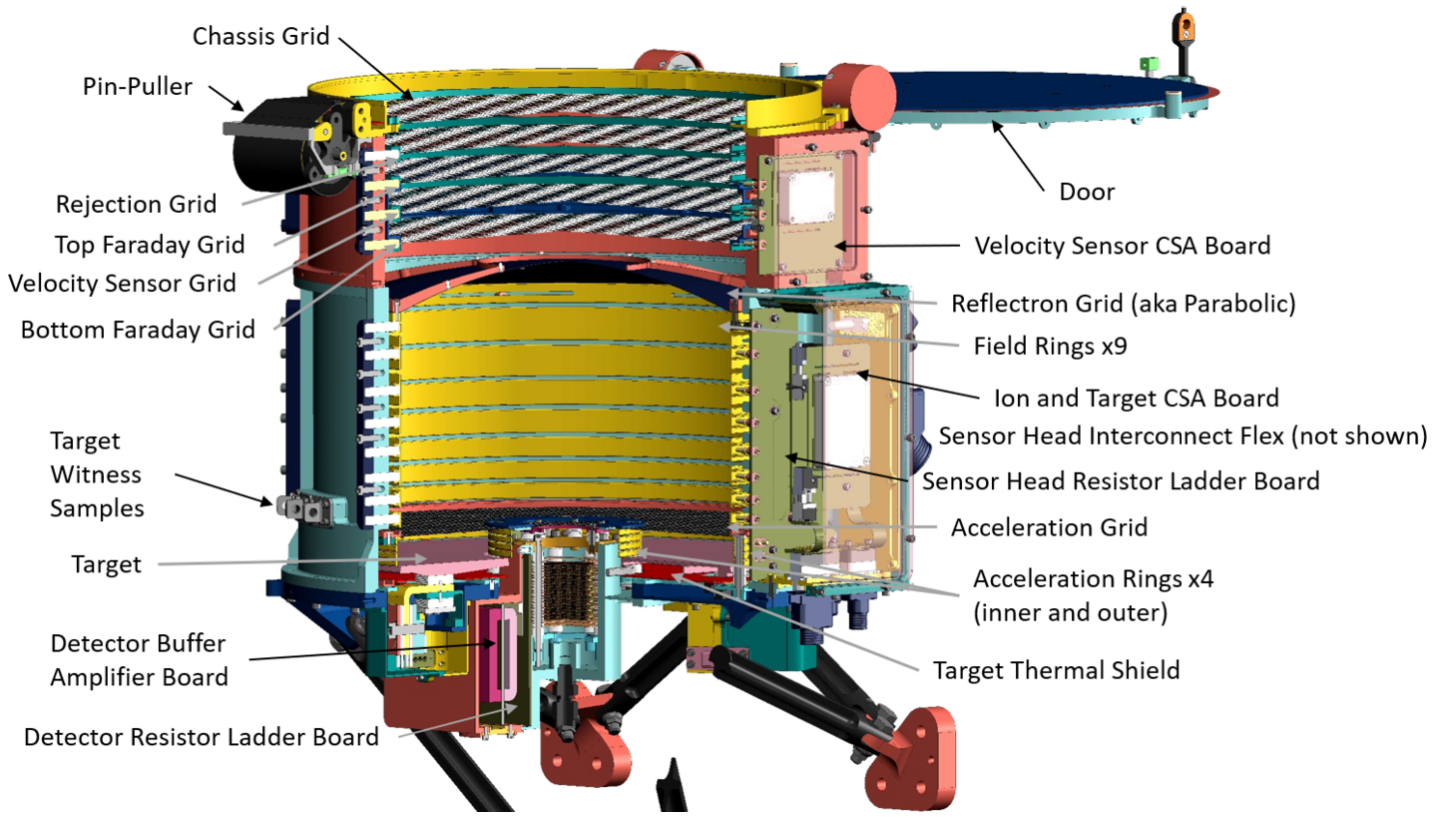
Cut-away view of the SUDA Sensor Head showing its components

The SUDA instrument is comprised of two distinct subsystems: The Sensor Head and the Remote Electronics Box (REB). The Sensor Head is affixed to the exterior of the spacecraft, where it is exposed to the incoming dust particles. The REB is situated within in the spacecraft’s interior radiation vault and provides instrument power regulation, data handling, and interface to the spacecraft. Intra-instrument cables connect the Sensor Head and the REB, routing power, analog science signals, high voltage, and thermal control. SUDA is designed for low mass and mechanical integrity during launch. The effects of the radiation environment on the Sensor Head are mitigated by limiting the number of electronic components to a minimum and by housing them in three radiation-shielded compartments (Fig. 7).

#### Sensor Head

The Sensor Head (Fig. 7) is composed of the Detector Assembly (Sect. 3.2.4), the Target Assembly (Sect. 3.2.3), the Ion Optics Assembly (Sect. 3.2.2), and the Velocity Sensor Assembly (Sect. 3.2.5), the Door Assembly (Sect. 3.2.6), and the mounting struts. The height of the Sensor Head is 560$~\mathrm {mm}$, including the mounting struts, and 351$~\mathrm {mm}$ without the struts. Its maximum width (parallel to the mounting interface) is 337$~\mathrm {mm}$, and its maximum depth (perpendicular to the mounting interface) is $320 ~\mathrm {mm} $. These dimensions encompass all the protrusions from the main cylinder, which has an outside diameter of 289$~\mathrm {mm}$. The DC magnetic moment of the Sensor Head is 21 $\mathrm{mA\,m^{-2}}$ (power-off: 8 $\mathrm{mA\,m^{-2}}$).

The SUDA Sensor Head is connected to the spacecraft via six titanium struts. The six struts are affixed to the spacecraft via three aluminum pads. Two struts connect to each mounting pad, and three screws connect each pad to the spacecraft structure. The end of each strut is terminated via a lug and clevis joint. The free rotational degree of freedom of the lug and clevis joints, in conjunction with the relatively low bending stiffness of the struts, results in a semi-kinematic mounting configuration. The struts are designed to thermally isolate the Sensor Head from the spacecraft, meet the less than 0.33^∘^ boresight to spacecraft panel alignment requirement, and react to mechanical loads during launch. Additionally, they are required to have a fundamental vibration frequency greater than 100$`\mathrm {Hz}$. During the flight model sensor head vibration test, the lowest observed frequency mode of vibration was 119$`\mathrm {Hz}$.

The mass of the Sensor Head is 9.6$~\mathrm {kg}$, including 0.95$~\mathrm {kg}$ for the aluminum and stainless steel radiation shielding. The breakdown of the sensor mass into the mass of the assemblies is detailed in Table 2.

**Table 2:** SUDA mass breakdown. Total and component masses are based on measurements of the SUDA flight model. Shielding mass is estimated and is the additional shielding mass needed for the Jovian environment as compared to more benign planetary radiation environments

System/assembly	Mass (g)	Est. shielding (g)
SUDA Instrument System	14,557	1985
SUDA Sensor Head Subsystem	9595	950
Detector Assembly	1030	430
Ion Optics Assembly & Electronics Enclosure	2494	250
Target & Acceleration Grid Assembly	2141	100
Velocity Sensor & Rejection Grid Assembly	1427	120
Door Assembly	806	0
Mounting Struts and Pads	725	0
MLI Blankets	715	0
Miscellaneous Sensor Head Components	257	50
SUDA Remote Electronics Box (REB) Subsystem	4175	915
SUDA Electronics Boards	1654	240
Enclosure Structure and Mounting Hardware	2521	675
SUDA REB to Sensor Head Harnesses	788	120
HV Harness	240	30
Analog Harnesses	234	40
Power Harnesses	314	50

#### Ion Optics Assembly

The SUDA ion optics represents a scaled version of the Large Area Mass Analyser (LAMA) (Srama et al. 2005), as previously developed for the proposed Cosmic DuneXpress dust telescope mission (Grün et al. 2009) and the SARIM sample return mission (Srama et al. 2009a). However, there are notable discrepancies between the SUDA and LAMA designs. The former does not feature a field-free drift region between the acceleration grid and the reflectron unit, while the latter employs a greater number of ring electrodes. The SUDA Ion Optics Assembly is comprised of the target and the acceleration grid, which are situated at the lower portion of the assembly, a parabolic grid at the upper portion, and ring electrodes. Two sets of four potential rings, one positioned on the target’s outer diameter and the other on its inner diameter, are employed to ensure uniformity of the electric field between the target and the acceleration grid at their respective edges. The deceleration region is situated between the acceleration grid and the parabolically shaped reflectron grid. The electric field between these two components is maintained at a uniform level by nine ring electrodes. The potential rings in both the reflectron unit and the accelerator region are gold-plated aluminum and are separated via ceramic spacers.

**Fig. 8:**
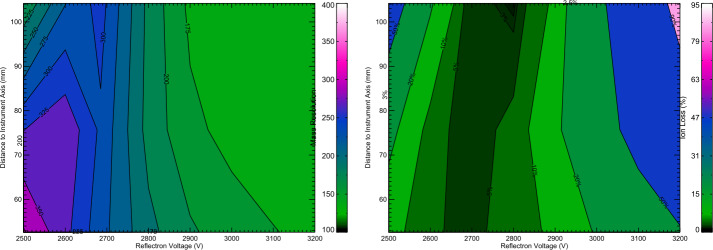
SIMION/SpecSim simulations of the SUDA ion optics performance for a 2$~\mathrm {eV}$ isotropic impact plasma typical for ice particle impacts. The ions are assumed to be released instantaneously, i.e. retardation effects leading to asymmetric line shapes as discussed in Sect. 4.1.4 have been ignored. **Left:** Color coded mass resolution as function of the reflectron voltage, $U_{\mathrm {ref}}$, and on the radial distance of the impact location to the SUDA symmetry axis, $r_{\mathrm {start}}$. **Right:** Dependence of the color-coded ion collection efficiency (the fraction of the launched ions arriving at the ion detector) on $U_{\mathrm {ref}}$ and $r_{\mathrm {start}}$. At $U_{\mathrm {ref}} \sim 2685 ~\mathrm {V} $, both the mass resolution and the ion collection efficiency are maximum and independent of $r_{\mathrm {start}}$

The field optics parameters, including the number, position, and potentials the of ring electrodes, the potential and shape of the parabolic reflectron grid, and the location of the ion sensor, have been optimized through an iterative process using the SIMION 8 ion trajectory software (Dahl 2000) and the in-house software SpecSim (Kempf et al. 2012b; Williams 2015; Levin and Kempf 2022). Utilizing the SIMION simulation data, SpecSim calculates the resulting TOF spectral line shapes and derives the corresponding mass resolution and ion-focusing parameters. The objective of the optimization process was to achieve a mass resolution exceeding 200 and a 100% ion collection efficiency for any impact location on the target (see Fig. 8). The simulations further demonstrated that minor discrepancies in the ion optics geometry (e.g., due to manufacturing tolerances) or slight fluctuations in the bias potentials (e.g., due to the High Voltage power supply precision and divider resistor component tolerance) have an insignificant impact on SUDA’s performance. The simulation outcomes align with the experimental data obtained at the Dust Accelerator Facility at the University of Colorado (Sect. 4).

#### Target Assembly

**Fig. 9:**
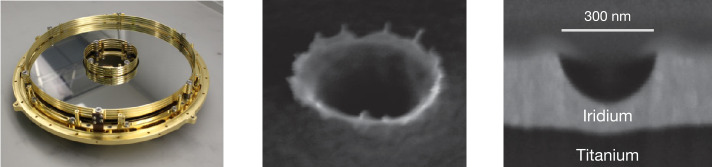
**Left:** The SUDA Target Assembly. **Middle:** SEM image of an crater created by a 5$~\mathrm {km\,s^{-1}}$ impact of a 200$~\mathrm {nm}$ iron particle onto a SUDA target verification sample. The thickness of the iridium layer on top of the titanium carrier is 250$~\mathrm {nm}$, the surface roughness of the iridium coating is 5$~\mathrm {nm}$-RMS. **Right:** Edge-on view of the same crater after an ion beam carved a groove through it. The impactor has not punched through the hard iridium coating but only plastically deformed the layer, implying that ions from the titanium carrier will not be present in the corresponding mass spectrum

The target is composed of commercially pure Grade 2 titanium and is coated with a 250$~\mathrm {nm}$ thick layer of high-purity iridium, which exhibits excellent impact ionization properties. Iridium is chemically inert, and its two isotope lines at 191$~\mathrm {u}$ and 193$~\mathrm {u}$ do not interfere with the mass lines of interest observed in water ice spectra. The high density and tensile strength of iridium enables a high impact charge yield. The iridium coating is applied via magnetron sputtering. The sputtering source material is 99.967% pure iridium, with the remaining impurities consisting of platinum (0.015%), rhodium (0.008%), and ruthenium (0.005%) (Table 3). Three ceramic heaters are mounted to the rear of the target and are capable of raising the temperature of the target to 120$~{}^{\circ }\mathrm {C}$ for the purpose of decontamination. The target assembly is thermally isolated with a decontamination heater affixed at the base. To minimize the target area exposed to contaminants as well as the heat loss during decontamination, the target surface has a surface roughness of only 5$~\mathrm {nm}$-RMS (Root Mean Square). The target assembly is thermally isolated from the surrounding instrument components by means of titanium mounting flexures and a heat shield. The iridium layer has been demonstrated to withstand impacts by dust analogs in the relevant speed and size range (Fig. 9), repeated thermal stresses induced by decontamination, and mechanical stresses due to vibrational loads.

**Table 3:** Chemical composition of the iridium coating

Element	%	Element	%	Element	%
Platinum	0.0145	Iridium	99.9672	Palladium	0.0007
Rhodium	0.0077	Ruthenium	0.0054	Gold	0.0005
Iron	0.0005	Copper	0.0005	Nickel	0.001
Lead	0.0002	Aluminum	0.0003	Silicon	0.001
Barium	0.0005				

The exceptional cleanliness of the target has been corroborated through impact experiments conducted at the IMPACT accelerator facility. Impact mass spectra of pure aluminum particles exclusively exhibit mass lines concordant with the target and projectile material, whereas typical contaminants, including sodium, potassium, and carbon, have not been identified.

#### Ion Detector Assembly

**Fig. 10:**
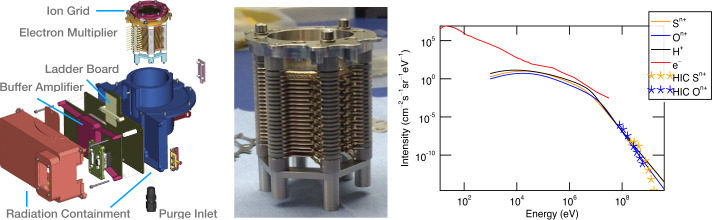
Ion Detector Assembly. **Left:** An exploded-view drawing of the assembly. **Middle:** The electron multiplier composed of 20 stacked dynodes operated as the TOF ion detector. **Right:** Energetic particle flux at Europa’s orbital distance. Charged particles with energies $< 3.2 ~\mathrm {keV} $ will be rejected by the instrument’s shielding grid and the reflectron grid. Particles with higher energies will hit the mutiplier and contribute to the detector’s background current by creating secondary electrons (one per particle or less)

The Ion Detector Assembly is situated at the base of the instrument, with its aperture extending into the center of the target area to enable the collection of ions from the ion optics. The ion detector is encased in a cylindrical compartment that provides radiation shielding and is attached to the detector electronics enclosure. The detector electronics comprise the Detector Ladder Board, which establishes the potentials of the individual dynodes via a resistor ladder, and the Detector Buffer Board, which accommodates the detector gain stage amplifier circuits and interfaces to the Remote Electronics Box (REB) through intra-instrument cables (Fig. 10). The ion grid is integrated into the front of the Ion Detector Assembly, allowing it to intercept a fraction of the ions before they reach the multiplier. The ion grid is protected by the acceleration grid and a shielding grid situated between the ion grid and the multiplier. A coaxial cable facilitates the connection between the ion grid and the ion grid CSA electronics. The detector housing is thermally coupled to the sensor head structure, whereas the detector buffer board is thermally isolated. The limiting aperture of the detector housing is defined by the circular ion grid clamp, which has an internal diameter of 30.5$~\mathrm {mm}$. The FOV of the detector is 30^∘^.

The ion detector utilized in SUDA is analogous to the MM1 electron multiplier from Johnston Technologies employed in Cassini CDA (Srama et al. 2004), and has been developed by LASP (Fig. 10 Middle). The detector is comprised of 19 stacked, flat, discrete dynodes, each etched to a shape that focuses the electrons from one dynode to the next, utilizing the applied potential. The top dynode functions as an ion-to-electron converter, with an efficiency of approximately 25%. The detector anode, situated below the bottom dynode, serves to collect all the electrons. This configuration results in the formation of 18 electron multiplication stages. The detector exhibits a response time of $\approx 15 ~\mathrm {ns} $ Full Width at Half Maximum (FWHM), which is sufficiently short to prevent any compromise to mass resolution. The MM1-type electron multiplier has been selected for its robust design, heritage, proven performance history, and high dynamic range. The detector is capable of a total gain $\sim 10^{8} $ and in SUDA it is operated with a gain of $10^{5}$. The front of the detector is connected to the chassis ground, and the anode is biased to a potential of +4$~\mathrm {kV}$. The amplified circuit is AC-coupled to the anode via a high-voltage blocking capacitor. The TOF signal, measured as current pulses to the anode, is amplified by a three-output gain stage amplifier to achieve the required high dynamic range (see Sect. 4.1.2).

#### Velocity Sensor

**Fig. 11:**
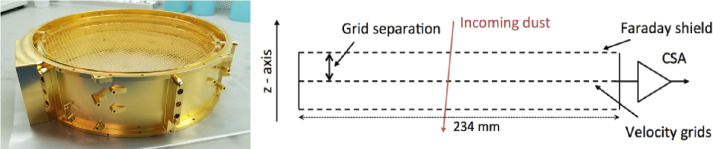
Velocity Sensor

The velocity sensor (Fig. 11) employs a charge pickup method to measure the velocity of incoming particles, a technique that has been demonstrated to be effective by Cassini/CDA (Auer et al. 2002; Kempf et al. 2004, 2006, 2023). The enhanced sensitivity observed in comparison to CDA is achieved through the utilisation of low-noise electronics situated in close proximity to the grid, which is encased in a Faraday shield. The Velocity Sensor Assembly represents the uppermost component of the Sensor Head and is comprised of five grids, as detailed in Sect. 4.2. The outermost grid is connected to the chassis ground, followed by the electrically isolated and biased rejection grid. The velocity grid is the fourth from the top and is mounted in between two electrically isolated grids in a double-walled subassembly that provides a complete Faraday shield connected to the analog ground of the CSA, which amplifies the induced charge signal. The Velocity CSA is encased in a radiation shielding box mounted on the side. The grids of the Velocity Sensor are photo-etched from 127$~\textrm {\textmu }\mathrm {m}$ molybdenum with an open area of 95%. The grids are separated by 20$~\mathrm {mm}$ and supported by titanium flexures to accommodate thermal expansion differences.

#### Door Assembly

The SUDA door is a one-time deployable cover that serves to mitigate contamination of the target and the ion detector during ground integration and test activities, as well as during launch and for a portion of the cruise phase. With the exception of ground calibration testing, thermal testing, and brief door functional tests, the door is to remain closed until the Europa Clipper spacecraft reaches 2$~\mathrm {AU}$. The Door Assembly is composed of a door, a spring-loaded hinge, a flexible aperture seal, a release mechanism, end-of-travel springs, open indicator switches, and a kick-off spring. Furthermore, the Door Assembly incorporates the outer grounded grid, which offers Faraday shielding for the rejection grid. The door assembly is affixed to the Velocity Sensor and Rejection Grid Assembly.

The release mechanism is an Ensign-Bickford Aerospace and Defence (EBAD, previously TiNi Aerospace) P10 Pin Puller actuator. The actuator incorporates redundant shape memory alloy components, enabling manual reset on the ground without the necessity for door disassembly, which facilitates functional testing. The door is constructed from aluminum and has been designed to be lightweight and to exhibit minimal rotational inertia. A thin (0.15$~\mathrm {mm}$) and flexible aperture seal, made of copper-clad polyimide, encircles the perimeter of the door. Its purpose is to restrict nitrogen purge gas and limit the ingress of particulate and molecular contamination during ground tests, launch vehicle integration, and ascent until the door is deployed (see Sect. 3.5). The deployment of the door is initiated by the application of strain energy within two redundant torsion springs located in the hinge. A kick-off spring, situated in close proximity to the actuator, provides supplementary deployment torque and facilitates the separation of surfaces in contact. The hinge is designed to rotate on a 316 stainless steel shaft via the use of redundant bushings made from molybdenum disulfide impregnated polyimide (DuPont Vespel SP-3). This results in the reduction of parasitic frictional torques in a vacuum environment. Additional bushings are situated between the shaft and the torsion springs, serving to reduce the friction between these two moving parts. The clearances and manufacturing tolerances between the shaft, bushings, and door permit operation of the door within a very wide temperature range, from $-90 ~{}^{\circ }\mathrm {C} $ to $+50 ~{}^{\circ }\mathrm {C} $. Subsequently, the door sweeps a 180^∘^ arc, making contact with titanium end-of-travel springs, which serve to mitigate the impact loads associated with reaching the end of its travel. As a consequence of the low-friction hinge, the door oscillates several times against the end-of-travel springs until it reaches a state of rest. In this deployed position, two redundant microswitches are contacted, which provides an open indication signal to the spacecraft. The torque from a single torsion spring in the hinge is sufficient to hold the door open against the end-of-travel springs when the spacecraft is subjected to the maximum expected acceleration. The total swept angle of the door from the stowed position to the deployed equilibrium position is approximately 195^∘^, which prevents the door from becoming a glint source for UV.

#### Remote Electronics Box

The Remote Electronics Box (REB), situated within the vault, contains the High Voltage Power Supply (HVPS), the Low Voltage Power Supply (LVPS) (each measuring 200$~\mathrm {mm}$ by 150$~\mathrm {mm}$), and the Processor Board (measuring 200$~\mathrm {mm}$ by 125$~\mathrm {mm}$). The boards are affixed to the housing using cardlocks, which are integrated into an aluminum enclosure with a flange for mounting. All connectors are accessible from a single side, and the connections between the boards are made via short rigid flex assemblies. The total volume of the Remote Electronics Box is 2600 $\mathrm{cm^{3}}$. The REB weighs 4.2$~\mathrm {kg}$, including spot shielding for the Field-Programmable Gate Array (FPGA). The cabling is 7.6$~\mathrm {cm}$ long and weighs 0.4$~\mathrm {kg}$. The DC magnetic moment of the REB is 30 $\mathrm{mA\,m^{-2}}$ (power-off: 17 $\mathrm{mA\,m^{-2}}$).

#### Thermal Design

The SUDA Sensor Head and REB have separate thermal interfaces to the Europa Clipper spacecraft. The Sensor Head is thermally isolated from the spacecraft via its titanium struts. A passive thermal design approach is employed for the Sensor Head structure, ion optics, and grids (as well as the Door Assembly subsequent to its deployment). The Sensor Head is insulated with the use of Multi-Layer Insulation (MLI) blankets. In order to minimize power consumption, only the components on the Sensor Head without sufficiently large operational and survival temperature ranges range are equipped with active thermal control mechanisms. These include the Velocity CSA, the Ion and Target CSA, the Detector Buffer, and the door latch mechanism. In the most adverse cold environment encountered during the science phase, an average of 1.7$~\mathrm {W}$ of operational heater power is required to provide supplementary heat to the Sensor Head electronics, thereby ensuring that their temperatures remain within the operational range ($\geq -40 ~{}^{\circ }\mathrm {C} $). The operational heaters are supplied by a SUDA LVPS regulated and filtered output, which serves to prevent the injection of noise from the spacecraft power lines into SUDA’s sensitive amplifier circuits. In the event that SUDA is powered off, the spacecraft-controlled survival heaters provide an average of $1.8 ~\mathrm {W} $ to maintain the electronics within their survival range ($\geq -50 ~{}^{\circ }\mathrm {C} $). A latch heater provides heat to the pin-puller actuator, thereby enabling a wide deployment range for the SUDA door.

The target decontamination heater is capable of drawing up to 40$~\mathrm {W}$ of power and is controlled in a bang-bang fashion via platinum resistance thermometers (PRTs) located within the Target Assembly. The temperature ramp is to 120$~{}^{\circ }\mathrm {C}$ controlled over a four hour period, during which an average of 16$~\mathrm {W}$ is consumed. Once the temperature has reached 120$~{}^{\circ }\mathrm {C}$, it is maintained for an additional four hours, which requires an average power consumption of 14$~\mathrm {W}$. During the decontamination dwell period, the mean temperature of the iridium-coated surface of the target is 120$~{}^{\circ }\mathrm {C}$. However, due to the low thermal conductivity of titanium and the target’s view to deep space, a temperature gradient exists across its surface with analytical temperatures predicts between 132$~{}^{\circ }\mathrm {C}$ and 116$~{}^{\circ }\mathrm {C}$. A cold spot, measuring approximately 1$~\mathrm {cm}$ by 3$~\mathrm {cm}$, exists where the target high voltage cable attaches at the outer diameter, with predicted temperatures as low as 105$~{}^{\circ }\mathrm {C}$.

The spacecraft is responsible for thermal control of the vault as a whole, regulating the base temperature of the electronics boxes within. The heat dissipated by the electronics within the REB is conducted through its aluminum structure to the spacecraft. The REB Processor Board (see Sect. 3.3.1) includes a braided copper thermal strap, which removes heat from the FPGA.

### Analog and Digital Electronics

#### Electronics Overview

**Fig. 12:**
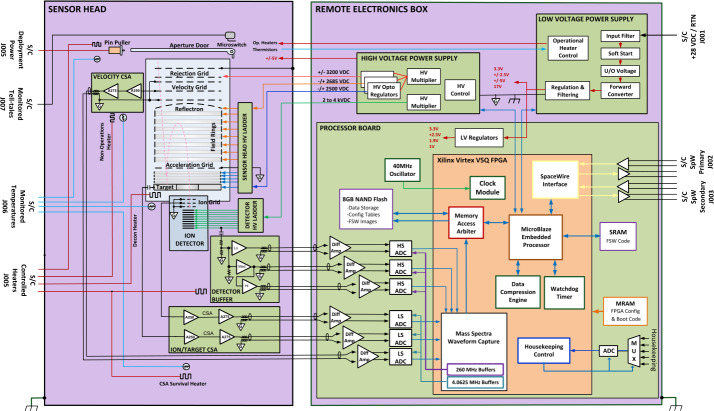
SUDA electronics block diagram

The SUDA electronics block diagram is illustrated in Fig. 12. The harsh Jovian radiation environment has necessitated the limitation of the electronics housed in the Sensor Head. These are restricted to the amplifiers required to read out and condition SUDA’s six analog science channels, as well as to filter and divide the high voltages supplied by the HVPS for the ion optics and ion detector. The Sensor Head contains five circuit boards: the Velocity CSA (VCSA), the Ion Grid and Target CSA (ITCSA), the Detector Buffer, the Sensor Head High Voltage Ladder, and the Detector High Voltage Ladder. A test connector on the Sensor Head (not shown in the block diagram) allows for the injection of test signals into each of the six amplifier circuits, thereby facilitating the ground testing of the instrument.

The Sensor Head has electrical interfaces with the spacecraft and REB. The spacecraft provides redundant deployment power for the door pin puller actuator and monitors the state of the door’s end-of-travel microswitches. Additionally, the spacecraft also provides target decontamination and survival heater power with temperatures being provided by spacecraft-monitored platinum resistance thermometers (PRTs). The REB is connected to the Sensor Head via six cable assemblies. The Power and Temperature Harness is responsible for supplying power to amplifier circuits, operational heater power, and instrument-monitored temperature measurements. The Analog Harness is responsible for routing the analog signals associated with the six science channels to the REB. Finally, four high-voltage coaxial cables facilitate the transmission of high-voltage from the HVPS. These high-voltage cables and their connectors were jointly developed with the Southwest Research Institute for use on both the SUDA and the Europa Clipper MASPEX instrument. Both the Power and Temperature Harness and the Analog Harness are connected to bulkhead connectors on the exterior of the spacecraft’s radiation vault. Harnesses located on the interior of the vault facilitate the connection between the bulkhead and the REB. The high-voltage cables traverse the interior of the vault without interruption and are connected directly to the REB via a radiation-shielded feed-through.

The REB is comprised of the LVPS, HVPS, and Processor circuit boards. The REB is situated within the radiation vault, which exhibits a relatively lower level of radiation and thermal exposure compared to the Sensor Head. The REB is equipped with a test port (not depicted in the block diagram), which facilitates ground testing through the provision of an RS-422 diagnostic interface, a high voltage inhibit signal for test safety, and triggering inputs and outputs that enable the capture of auxiliary test data during calibration. During flight, test port interfaces are disabled by the FPGA. The REB receives unregulated power from the spacecraft, and redundant SpaceWire interfaces provide SUDA’s command and data handling connection to the spacecraft.

SUDA employs a multi-point grounding methodology to attain low-noise functionality for the Sensor Head’s analog science channels and to ensure immunity to the anticipated radiated and conducted electromagnetic interference (EMI) environment during flight, including electric fields from the REASON instrument and Europa Clipper’s ice-penetrating radar. The REB has a single-point connection to the spacecraft chassis ground at the LVPS. In contrast, the Sensor Head employs multiple chassis ground connections. The Velocity CSA, the Ion and Target CSA, and the Detector Buffer boards each have a direct low impedance connection to the chassis. This grounding configuration was determined through the implementation of conducted susceptibility and instrument performance testing on the SUDA engineering model with various grounding configurations. The configuration that met the requirements and exhibited the best overall performance was selected.

#### Sensor Head Electronics

##### Velocity Grid CSA Board

The VCSA is responsible for amplifying the signals that are measured by the Velocity Grid electrode. The circuit employs an Ametek (formerly Amptek) A250F charge-sensitive preamplifier to convert charge into a voltage signal, which is subsequently amplified by an Ametek A275FN pulse amplifier. Both the A250F and the A275FN are hybrid microcircuits. The differential signals are injected into the analog harness via a balun transformer. The analog front-end of the Processor Board buffers the VCSA signal and provides anti-alias filtering prior to digitization by a low speed ADC. Diodes are employed to safeguard the amplifiers from the potential damage caused by electrostatic discharge (ESD). The VCSA circuit is designed to reproduce the triangular image charge waveform of a dust particle as it traverses the velocity grid over a speed range of 3.5 to $\sim 25 ~\mathrm {km\,s^{-1}} $ for particles with charges as low as 0.25$~\mathrm {fC}$ (see Sect. 4.2). Over the aforementioned speed range, the phase shift of the VCSA output is deemed to be insignificant negligible. The amplitude of the triangular waveform in relation to the baseline is indicative of the charge of the particle. The VCSA is the most sensitive SUDA amplifier, exhibiting a range of ±20$~\mathrm {fC}$. The VCSA gain calibration is outlined in detail in Sect. 4.2.

##### Ion Grid and Target CSA Board

The ITCSA comprises two discrete CSA circuits. The Target CSA is responsible for measuring the impact charge collected by the target, while the Ion Grid CSA is tasked with measuring the ions collected by the ion grid, which is situated directly in front of the ion detector. As with the VCSA, both ITCSA circuits employ the Ametek A250F and A275FN, yet both of these circuits integrate the measured charge. The discharge time constant of the integrator is considerably longer than the time required for the impact charge collection and the flight time of the heaviest ions. Similarly, as with the VCSA, balun transformers are employed to generate differential output signals, which are then transmitted to the REB via the analog harness. The analog front-end of the Processor Board employs identical buffering and filtering techniques as those utilized in the VCSA. The diode input protection circuits for ITCSA circuits provide protection from both electrostatic discharge (ESD) events and high-voltage partial discharge events. The Target CSA is capable of rise times of less than 2$~\mathrm {\textrm {\textmu }s}$, with a range of approximately ±2.5$~\mathrm {pC}$. After digitization, the Target CSA Digital Number (DN) to charge conversion is 3.9$~\mathrm {DN/fC}$. The Ion Grid CSA is capable of rise times of less than 1$~\mathrm {\textrm {\textmu }s}$, with a range of approximately $\pm 500 ~\mathrm {fC} $, and its conversion is 0.79$~\mathrm {DN/fC}$.

##### Detector Buffer Board

The high dynamic range TOF ion detector output is achieved by a three-channel gain stage amplifier circuit, which serves to amplify pulses from the detector anode. The Detector Buffer Board employs three Texas Instruments LMH6702 wideband operational amplifiers to generate three discrete TOF ion detector output signals with relative gains that are separated by factors of approximately 10 and 100, respectively, relative to the lowest gain channel. Differential transmission signals are generated through the use of balun transformers. The Processor Board then buffers the signals and performs band-pass filtering (15 to 135$~\mathrm {MHz}$).

##### High Voltage Ladder Boards

The Sensor Head is comprised of two high-voltage ladder boards, the Sensor Ladder Board and the Detector Ladder Board. The Sensor Ladder Board employs high-voltage divider resistors to generate intermediate voltages for the ring electrodes situated within the reflectron region and the acceleration region of the ion optics. Similarly, the Detector Ladder Board performs the same function for the detector dynodes. Furthermore, the Detector Ladder accommodates storage capacitors for the eight dynodes in closest proximity to the anode, which assist in preventing voltage drops between dynodes for large current pulses. The ladder boards include low-pass filters for each of the high-voltage inputs.

#### Signal Processing Electronics

The SUDA Processor Board, located within the REB, is responsible for signal processing, command and data handling operations for the instrument. It contains a Xilinx Virtex-5QV reconfigurable Field-Programmable Gate Array (FPGA) microcircuit, comprising $20{,}480$ configurable logic blocks (containing $131{,}072$ logic cells), $29{,}836$ kb RAM blocks ($10{,}728$ kb), and 836 I/O pins. The FPGA meets the mission design requirements by tolerating at least a total ionizing dose (TID) of 1$~\mathrm {Mrad(Si)}$ (Si). The SUDA Flight Software (FSW) is executed on a 40 MHz 32-bit MicroBlaze core embedded within the FPGA logic (see Sect. 3.4). The FPGA configuration images, amounting to 6 MB, are stored in a radiation-hard magnetic RAM (MRAM) (Honeywell HXNV06400 $4 \times 16$ Mb multi-chip module) which is capable of withstanding a TID of 1$~\mathrm {Mrad(Si)}$. The FPGA is clocked by a 40 MHz oscillator with frequency stability of 65 ppm over its full operational temperature range (SUDA will operate within a much narrower range) and of 10 ppm per year maximum aging. Phase-locked loops within the FPGA utilize the 40 MHz reference close to generate other clock frequencies.

The FPGA is continuously sampling the output from the TOF gain stage (Sect. 3.1.1, 4.1.2 at 260 MHz by providing an interface to three external 12 bit ADC channels via two dual-channel Texas Instruments ADC12D1620QML ADCs. Moreover, the output of the target (QT), ion grid (QI), and velocity grid (QV) amplifiers is continually sampled with 12 bit resolution at 4.0625 MHz (Sect. 3.1.1). After receiving a trigger (see Sect. 5.1.3), the sampled TOF waveforms are stored with 10 bit resolution per sample in discrete First-In-First-Out (FIFO) buffers, comprising four 1k$\times 108$ bit FPGA RAM blocks. The maximum length of a TOF waveform that can be stored is $31.5 ~\mathrm {\textrm {\textmu }s} $. Similarly, low-speed waveforms are stored with 12 bit resolution and a maximum length of 126$~\mathrm {\textrm {\textmu }s}$ in low-speed FIFOs. Once the recording has concluded, the FIFO data is transmitted via a dedicated wishbone bus to the FPGA’s NAND flash controller, which manages SUDA’s 8 GB non-volatile NAND flash memory, sized to accommodate up to $103{,}179$ events. After worst-case degradation due to radiation effects, the NAND flash memory is estimated to retain the capacity to store $21{,}952$ events at the conclusion of the mission.

In addition to the acquisition of science data, the FPGA performs a number of crucial instrument functions. To facilitate the lossless compression of recorded waveforms, a Rice compression engine (Rice and Plaunt 1971) has been implemented into the FPGA. The data stored in the NAND flash is stored uncompressed. The compression engine is employed when data is packetized for transmission by the spacecraft. The FPGA is also responsible for the state-of-health housekeeping collection, the control of the high-voltage and low-voltage power supplies, and the communication with the spacecraft via a redundant SpaceWire interface.

#### Power Supplies

The Low Voltage Power Supply (LVPS) board converts 32.8$~\mathrm {V}$ (nom.) from the spacecraft to $+1 ~\mathrm {V} $, $+1.9 ~\mathrm {V} $, $-2.3 ~\mathrm {V} $, $+2.5 ~\mathrm {V} $, $+3.3 ~\mathrm {V} $, $\pm 5 ~\mathrm {V} $, $\pm 6 ~\mathrm {V} $ and $+16 ~\mathrm {V} $ DC used by the instrument. The use of isolating switched mode converters in conjunction with linear regulators allows for the optimization of both efficiency and the tight regulation of output voltages. Local filters, especially at high speed circuits, serve to prevent the degradation of the DC signal throughout the system resulting from the rapid switching of currents. In order to minimize losses, the $1 ~\mathrm {V} $ and $1.9 ~\mathrm {V} $ supplies are situated on the Processor Board.

The High Voltage Power Supply (HVPS) provides DC high voltages to the rejection grid, reflectron, target, and ion detector. The polarities of the reflectron, target, and rejection grid bias voltages can be switched via a ground command to record cation/anion spectra. The HVPS employs a pair of transformers and two independent voltage multipliers to generate voltages reaching up to $+ 4000 ~\mathrm {V} $ for the ion detector and up to $\pm 3400$$~\mathrm {V}$ for the reflection, target, and rejection grid. The latter’s dual polarity is achieved through the use of parallel voltage multipliers, comprising one that generates a positive voltage and another that generates a negative voltage of equivalent magnitude. Six Southwest Research Institute SW1502 high-voltage opto-isolators (two on each of the three dual-polarity outputs) are employed for the switching of polarities and the independent regulation of the reflectron and target voltages. The detector supply voltage can be adjusted from $+200 ~\mathrm {V} $ to $+4000 ~\mathrm {V} $, the rejection grid from $\pm 200 ~\mathrm {V} $ to $\pm 3400 ~\mathrm {V} $, the reflectron from $\pm 200 ~\mathrm {V} $ to $\pm 2900 ~\mathrm {V} $, and the target $\pm 200 ~\mathrm {V} $ to $\pm 2600 ~\mathrm {V} $. The HVPS design necessitates that the absolute value of the rejection grid voltage exceeds that of the reflectron and target voltages. Logic embedded within the FPGA ensures that the polarity of the rejection grid is opposite of the reflectron and target. Ripple in the high-voltage outputs, with an amplitude of approximately 10$~\mathrm {mV}$, is mitigated by low-pass filters.

#### Power Requirements

The power necessary for SUDA operation is determined through laboratory assessments employing the SUDA Flight Model. The total power demand in the *Flyby Mode* (Sect. 5.1.4) is $18.6 ~\mathrm {W} $.Table 4 presents the SUDA power allocation by operations mode.

**Table 4:** SUDA average power by mode based on measurements of the flight model instrument. Power dissipation of the electronics is a function of temperature and is expected to vary by -5% to +10% with respect to these numbers during the mission. Heater power estimates are based on correlated thermal modeling results. Modes in which SUDA is off and spacecraft-controlled heaters are enabled are indicated. Decontamination and Warm Target modes include $1.8 ~\mathrm {W} $ of survival heater power for the Sensor Head electronics

Mode	REB electronics (W)	Sensor head electronics (W)	Electronics subtotal (W)	Sensor head heaters (W)	Total (W)
Boot	7.3	0.5	7.8	1.5	9.3
Idle/Safe	7.4	0.5	7.9	1.5	9.5
Flyby	15.9	1.3	17.1	1.5	18.6
Survey	12.5	1.3	13.8	1.5	15.3
Processing	7.3	0.5	7.8	1.5	9.3
Survival (off)	0.0	0.0	0.0	1.8	1.8
Decontam. (off)	0.0	0.0	0.0	16.6	16.6
Warm Target (off)	0.0	0.0	0.0	3.9	3.9

### Flight Software

**Fig. 13:**
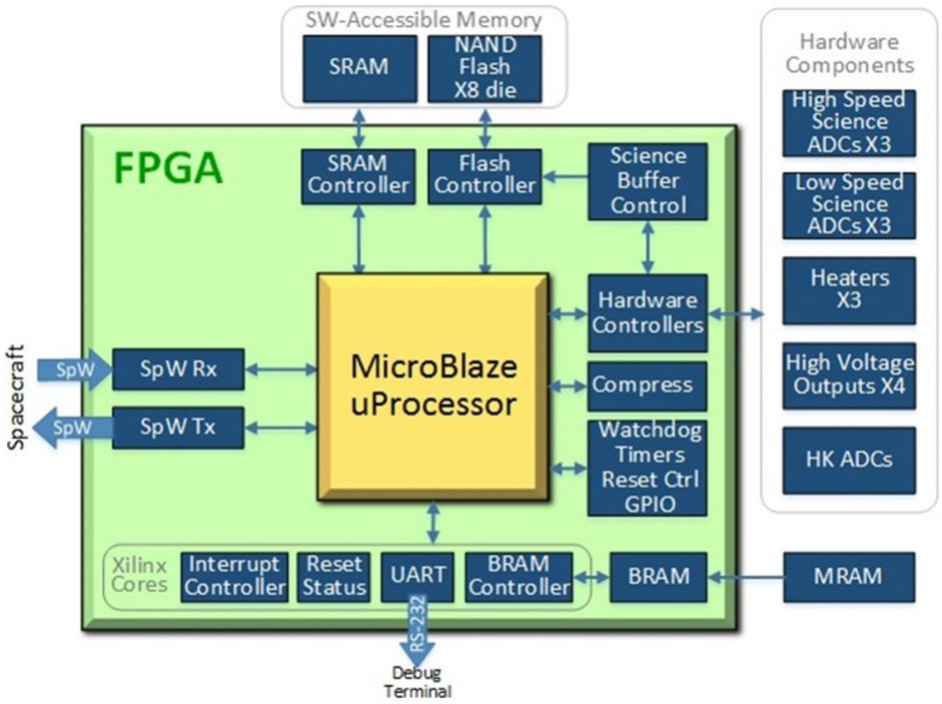
SUDA Flight Software

The SUDA C++ Flight Software (FSW) runs in the 32 bit Xilinx MicroBlaze processor embedded in the FPGA (Sect. 3.3.3). The SUDA FSW is responsible for executing commands for the SUDA instrument, maintaining its operational safety, directing the instrument to acquire and process scientific data, and determining which events to packetize for downlink (Fig. 13). The FSW is comprised of two distinct components: the Boot FSW and the Operational FSW. The primary responsibility of the Boot FSW is to locate a valid copy of the Operational FSW, either stored in non-volatile memory or received from the spacecraft, and then initiate its execution. The Operational FSW, in turn, provides comprehensive instrument functionality and can be modified or replaced at any stage of the mission.

In lieu of an operating system, both SUDA FSW components utilize a Deterministic Executive Scheduler (DES) to execute tasks at scheduled intervals. The majority of operations are conducted within tasks, encompassing memory management, command validation, telemetry generation, science data processing, fault detection and correction, and the onboard execution of stored command sequences (macros), which facilitate substantial instrument autonomy.

### Contamination Control

The contamination-sensitive components in SUDA are the impact target and the ion detector. The primary concerns regarding contamination control in SUDA are the deposition of molecular matter on the target and the accumulation of airborne salts in the ion detector prior to launch. Hydrocarbons present on the target have the potential to manifest as contaminants in the mass spectra, thereby impairing the performance of the mass spectrometer.

A variety of design strategies were employed to ensure the cleanliness of SUDA. Upon completion of the construction phase, the relevant contamination control procedures were duly followed, and a dependable witness plate program was put in place to confirm cleanliness during the ground testing phase.

The use of low-outgassing materials was a key aspect of the design, particularly in the Sensor Head. Even materials that are commonly used in aerospace applications were excluded from the design to guarantee self-compatibility. The use of epoxies and adhesives was employed at the subassembly stage, where feasible, with the objective of minimizing cure time in the vicinity of the target and detector. All materials underwent a vacuum bakeout prior to integration into the Sensor Head and the REB, with the Residual Gas Analyzer (RGA) employed to ascertain the requisite criteria for successful integration. In addition to the flight hardware, meticulous attention was dedicated to the selection and testing of Ground Support Equipment (GSE) materials, which were subjected to a vacuum bakeout and evaluated in a vacuum environment alongside the sensor head.

SUDA employs a gaseous nitrogen (GN2) purge until the launch phase (T-0), and budgetary provisions are made for potential outages at each stage of the ground campaign. The purge was initiated on the detector at the point of assembly of the dynode stacks. The T-0 purge on the Sensor Head was established with the integration of the target assembly. The maximum duration of purge outages is limited to 90 minutes, and a minimum four-hour on-purge period is required thereafter to allow particulates to return to pre-outage levels. The purge gas is an ultra-pure nitrogen that has been treated with optical purifiers prior to entering the Sensor Head. The purge design enters the system via the ion detector, subsequently diffusing outwards through the various ascent vent paths and Kapton seal around the door aperture. The flow rate is sufficient to ensure an effective exchange of nitrogen, thereby reducing the potential for particulate fallout within the sensor cavity.

Following vacuum baking and storage in controlled environments (ISO 6 or 7 clean rooms) until integration, the instruments were integrated in an ISO 7 clean room with minimal purge outages. During periods of inactivity, the Sensor Head was covered with an electrostatic discharge (ESD) safe bag, thereby enhancing the efficacy of the purge and minimizing the particulate fallout.

The transportation of SUDA to disparate test facilities throughout the instrument-level test campaign at LASP, along with the subsequent delivery of SUDA from Colorado to JPL, necessitated the implementation of specialized contamination control measures. A custom-fabricated shipping container was utilized to minimize the diffusion of contamination into the instrument. Prior to transportation or shipping, the air within the container was displaced by ultra-pure GN2. The pressure within the container was allowed to equalize via a one-way over-pressure valve and a one-way under-pressure valve, which facilitated the pull of outside air through an Entegris GateKeeper gas purifier.

To guarantee the mandatory level of cleanliness, a target witness program has been implemented to oversee the occurrence of significant contamination incidents up until the conclusion of launch preparations. To this end, nine iridium-plated witnesses have been manufactured for each target and stored in the closest possible proximity to the target (one for storage monitoring, three as controls for TOF-SIMS verification, and five witness targets for the sensor head monitoring). For the location of the witnesses, consult Fig. 7. One witness will be removed from the sensor in proximity to launch and stored for the purpose of identifying potential contaminants present in spectra of Europa surface material. The instrument’s cleanliness will be maintained by the protective door and the instrument’s continual purge until launch.

## Instrument Performance and Calibration

SUDA was subjected to rigorous testing and calibration at the IMPACT Dust Accelerator Facility at the University of Colorado (Shu et al. 2012). The 3 MV electrostatic accelerator emits single charged particles into a beam line comprising three mirror charge pickup tube detectors (Srama and Auer 2008), which measure the electrostatic charge $Q_{d}$ and the speed $v_{d}$ of the emitted particles in flight. Knowledge of $Q_{d}$ and $v_{d}$ readily provides the dust mass via

1 $$ m_{d} = (2 Q_{d} U_{acc} / v_{d} ^{2})^{1/2}, $$

where $U_{acc}$ is the accelerator potential. The dust beam has been focused on SUDA, which has been housed in a large vacuum chamber. The three pickup tube signals were recorded with a LeCroy oscilloscope triggered by the SUDA FPGA. This procedure allows us to associate the recorded SUDA events to be unambiguously associated with $Q_{d}$ and $v_{d}$ deduced from the pickup tube signals.

**Fig. 14:**
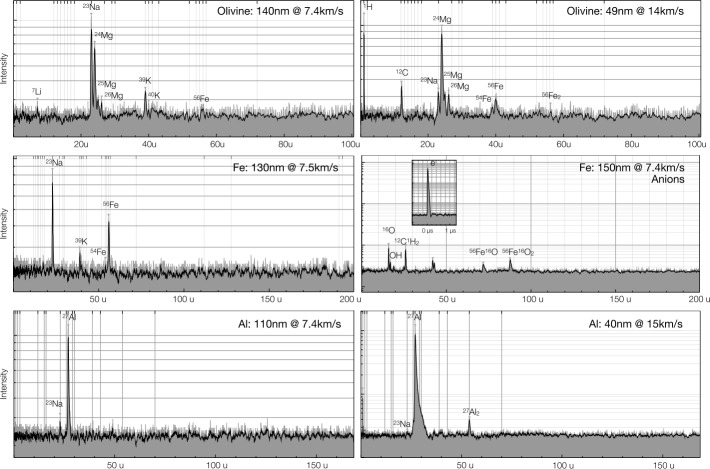
Examples of mass spectra recorded with the SUDA FM. **Top Row:** Cation spectra of olivine particle impacts recorded in Campaign 1. **Middle Row:** Cation and anion spectra of 7.5$~\mathrm {km\,s^{-1}}$ iron particle impacts. The electron mass line in the anion spectrum (composed of 99.8% of the ions appearing in the spectrum) is to narrow to be visible when plotted in the mass domain. The inset shows the electron peak in the time domain. **Bottom Row:** Cation spectra of aluminum particles recorded in Campaign 4

To verify the SUDA performance, microscopic iron, aluminum, and platinum coated olivine particles were employed as dust analogs. Table 5 provides an overview of the four dust impact campaigns. Fig. 14 shows a few examples of spectra recorded with the SUDA Flight Model (FM). We used iron because these materials have been employed for performance testing by all dust detectors before SUDA (Göller and Grün 1989; Srama et al. 2004; Horányi et al. 2014a). Aluminum was selected due to its relatively low density, which permitted the testing of the SUDA velocity sensor over a broad range of sizes and speeds (see Sect. 4.2). The mineral olivine is an optimal material for evaluating SUDA’s capacity to ascertain isotopic ratios of species in an impact mass spectrum (see Sect. 4.1.4). At the time of the SUDA calibration campaign, the technology for accelerating microscopic ice particles was not yet available. As of 2024, the IMPACT facility is in the process of commissioning an ice particle accelerator developed by a group at the University of Leipzig, Germany (Spesyvyi et al. 2024). This will be available for calibration runs with the SUDA Engineering Model (EM) at the end of 2025.

**Table 5:** SUDA Flight Model calibration campaigns at the CU Boulder/IMPACT dust accelerator facility (Shu et al. 2012)

Campaign	Date	Material	Number of impacts			
			Total	Cation	Anion	Grid/wall
1	December 2021	Olivine	290	159	131	0
2	March 2022	Aluminum	314	314	0	0
3	July 2022	Aluminum	2083	1957	126	81
4	August 2022	Iron	1993	1656	337	12

**Fig. 15:**
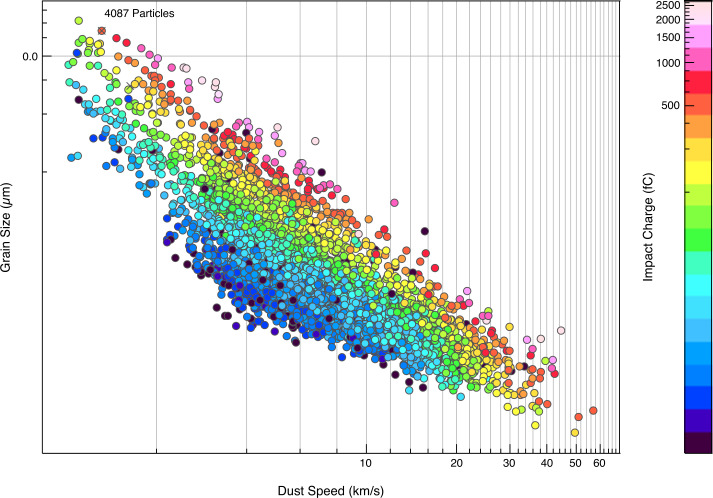
The mass and speed distribution of the SUDA FM calibration data set. The apparent correlation between the mass and speed of the detected grains results from the fact that the particles acquire similar kinetic energies from the electrostatic accelerator. The color code provides insight into the plasma charge generated upon impact. The observation of parallel linear structures in the log-log plot of $q_{imp}$ suggest a power law dependence of $q_{imp}$ on $m_{d}$ and $v_{d}$

The total number of recorded dust impacts was 4680 dust impacts, including impacts onto the instrument walls, grids, and the ion detector (see Table 5). Fig. 15 depicts a scatter plot of the speeds $v_{d}$ and masses $m_{d}$ of the detected particles. The size range of $22 ~\mathrm {nm} \le r_{d} \le 2.7 ~\mathrm {\textrm {\textmu }m} $ and speed range of $1 ~\mathrm {km\,s^{-1}} \le v_{d} \le 57 ~\mathrm {km\,s^{-1}} $ encompasses the range of particle sizes and velocities relevant for impacts of Europa ejecta as well as of Galilean ring particles and even of impacts of interplanetary and interstellar dust particles during the mission’s cruise phase.

### Mass Spectrometer

#### Mass Scale

The mass scale calibration is accomplished by associating the masses of known atoms or molecules with the appropriate peaks in the TOF spectrum. The relationship between an ion’s flight time, $t_{i}$, and its mass, $m_{i}$, is

2 $$ t_{i} = t_{0} + a \sqrt{m_{i}}. $$

**Fig. 16:**
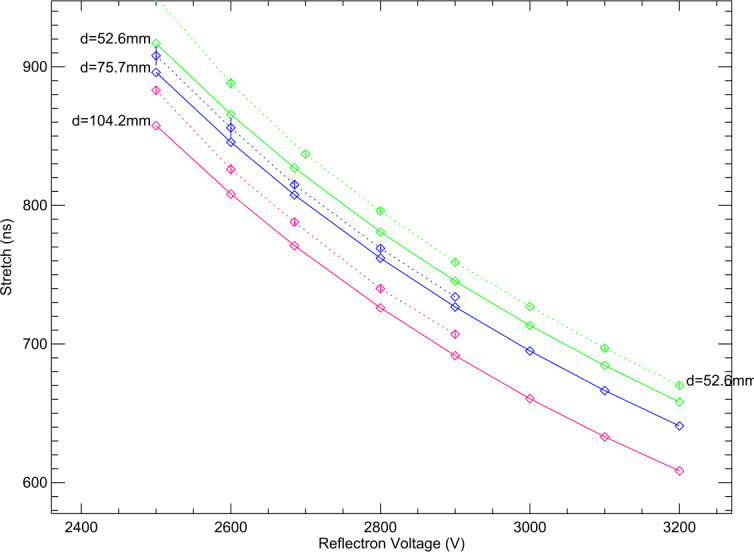
Dependence of the SUDA stretch parameter $a$ on the radial distance to the target center $r_{\mathrm {start}}$ and on the reflectron voltage $U_{\mathrm {ref}}$. Measured stretch parameters are marked with broken lines, solid lines are predictions from SpecSim/SIMION ion optics simulation (see Sect. 3.2.2). The presented data refers to the nominal SUDA acceleration voltage of $U_{acc} = 2500 ~\mathrm {V} $

Here, $a$ is the spectrum’s stretch parameter, which is derived from a fit to the set of $n$ identified mass lines $\{t_{i}, m_{i}\}_{i=1\ldots n}$. The shift parameter, $t_{0}$, is the initially unknown start time of the ions. In a second step, the masses of the unidentified peaks are calculated using Eq. (2) and the derived $a$ parameter. The stretch parameter is dependent upon the extraction voltage, $U_{acc}$, the reflection voltage, $U_{\mathrm {ref}}$, as well as on the radial distance of the impact site to the target center $r_{\mathrm {start}}$. Ions produced by impacts on the outer part of the target have longer flight paths to the ion detector than ions generated by impacts close to the target center, resulting in TOF spectra with a larger $a$ parameter. This is illustrated in Fig. 16, which depicts the measured dependence of $a$ on the distance, $r_{\mathrm {start}}$, of the ions’ start position to the instrument axis for various values of the reflectron voltage. Indeed, the dependence of the stretch parameter on $r_{\mathrm {start}}$ is advantageous for a flight instrument, as it allows for the impactor’s directionality to be constrained by the TOF spectrum itself. This is of particular importance for impacts of tiny grains whose charge insufficient for detection by the Velocity Sensor unit.

The SUDA mass spectrometer can be operated at any reflectron voltage, provided that the potential difference $U_{\mathrm {ref}} - U_{acc} $ exceeds the impact plasma’s temperature, $U_{imp}$, which is typically on the order of several$~\mathrm {eV}$. As evidenced by the simulations conducted using SpecSim/SIMION (see Sect. 3.2.2), the optimal reflectron voltage for the instrument’s ion optics performance is $2685 ~\mathrm {V} $ (at a nominal $U_{acc}$ = 2500$~\mathrm {V}$). This finding aligns with the calibration data.

#### High Dynamic Range (HDR) Mass Spectra

The complex organics spectrum depicted in Fig. 3 is a realistic illustration of an impact mass spectrum of an organic-laden water ice particle originating from the surface of Europa. The ratio between the weakest organic mass line that is relevant for the SUDA science case, $\mathrm {Tyr-H^{+}}$, and the strongest peak in this spectrum, $\mathrm {(H_{2} O)_{2} H^{+}}$, is approximately 260. In order to resolve the shape of a mass line, it is necessary to have at least five discrete amplitude steps. Therefore, the spectrum must be discretized with at least 1300 amplitude increments. Moreover, the amplitudes in an impact spectrum are proportional to the impact charge, which in turn is proportional to the grain mass (see Sect. 4.3). To record the reference mass spectrum for impactor masses varying by a factor of 20, a dynamic range of a few $10^{5}$ is required, which is not feasible with single-stage discretizers, such as those employed in the LDEX instrument on LADEE (Horányi et al. 2014b).

In order to enable the requisite wide dynamic range recording of the TOF spectrum, the signal at the ion detector’s anode is recorded with three distinct amplifier gains (for further details, refer to Sect. 3.3.2) and is then discretized with 10 bit resolution (Table 6). To address baseline drifts resulting from radiation exposure and saturation effects, the amplifier baseline has been set to the midpoint of the amplifier range, thereby achieving an effective resolution of 9 bits. The gain-corrected waveforms are then merged into a single High Dynamic Range (HDR) waveform with a maximum dynamic range of approximately $50{,}000$.

**Table 6:** SUDA Gain Stage parameters. The conversion between Digital Number (DN) and amplitude is $1\,\mathrm{DN}/ ~\mathrm {mV} $

		Sampl. time	Resolution	Gain	Clipping	
					Waveform	HDR
High Gain	TOF-H	3.84$~\mathrm {ns}$	10 bit	96	512$~\mathrm {mV}$	512$~\mathrm {mV}$
Mid Gain	TOF-M	3.84$~\mathrm {ns}$	10 bit	6	512$~\mathrm {mV}$	3072$~\mathrm {mV}$
Low Gain	TOF-L	3.84$~\mathrm {ns}$	10 bit	1	512$~\mathrm {mV}$	49,152$~\mathrm {mV}$

**Fig. 17:**
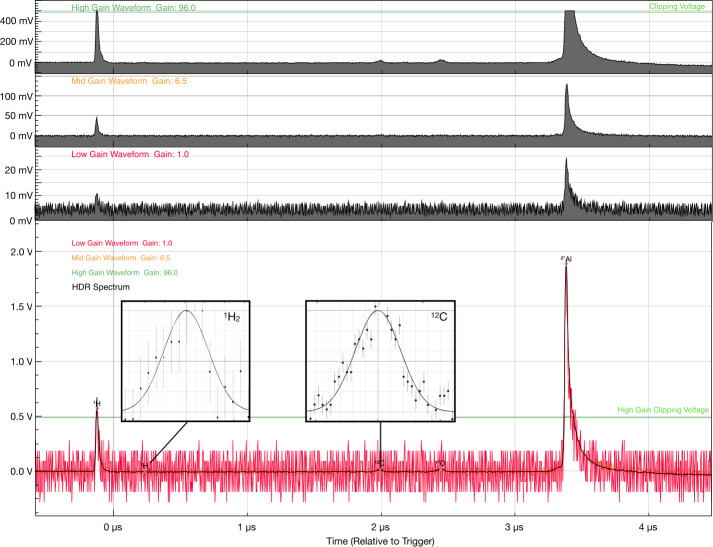
SUDA High Dynamic Range (HDR) spectrum of an 84$~\mathrm {nm}$ aluminum particle at 21$~\mathrm {km\,s^{-1}}$. The three top panels show the ion detector amplitude recorded with 3 different gains. The weak $\mathrm {^{1}H_{2}}$, $\mathrm {^{12}C}$, and $\mathrm {^{16}O}$ lines appear only in the high gain recording, while the strong $\mathrm {^{27}Al}$ is only fully resolved in the mid and low gain recordings. The bottom panel shows the gain corrected waveforms plotted together with the corresponding HRD spectrum (black line). The two insets show the faint $\mathrm {^{1}H_{2}}$ and $\mathrm {^{12}C}$ mass lines appearing in the recorded spectrum

To illustrate, Fig. 17 depicts an HDR spectrum of a fast aluminum particle impact, which resulted in an impact charge of $\sim 500 ~\mathrm {fC} $. The CDA mass spectrometer was unable to record mass spectra of such strong impacts due to saturation effects (Srama et al. 2004). The most intense line in the spectrum, $\mathrm {^{27}Al}$, exhibits a peak amplitude of $V_{p} = 2.05 ~\mathrm {V} $. The spectrum also displays faint lines, including those of $\mathrm {^{1}H_{2}}$ ($V_{p} = 6.5 ~\mathrm {mV} $), $\mathrm {^{12}C}$ ($V_{p} = 21 ~\mathrm {mV} $), and $\mathrm {^{16}O}$ ($V_{p} = 27 ~\mathrm {mV} $). These can only be resolved in the high-gain signal and are barely discernible when plotted on a linear scale. The amplitude ratio between the strongest and the weakest fully resolved lines is approximately 310, while the ion count ratio is approximately 625.

#### Mass Resolution

**Fig. 18:**
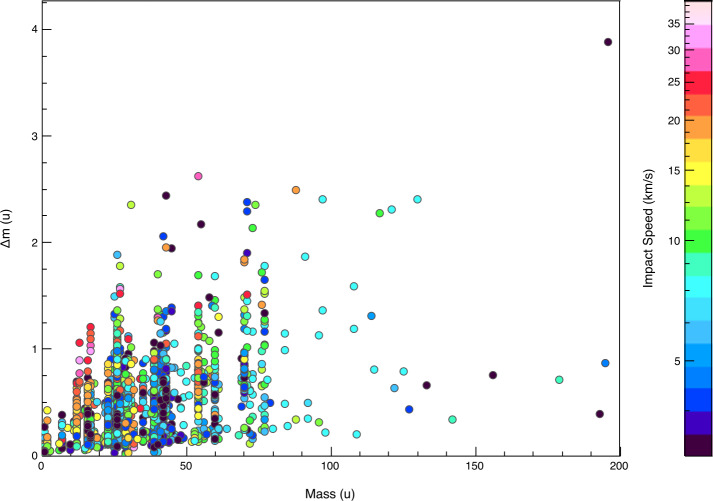
Line width $\Delta m$ as function of the ion molecule mass $m$ derived from line shape fits of SUDA FM mass spectra. The majority of the lines are narrower than $1 ~\mathrm {u} $ and can be resolved from adjacent integer mass lines

SUDA is required to resolve integer mass lines for ion molecule masses $< 200 ~\mathrm {u} $ (SUD.005 in Table 10), which implies that the Full Width Half Maximum (FWHM) line width in the mass domain must be $\Delta m \le 1 ~\mathrm {u} $ (Fig. 18). The majority of the mass lines in a representative subset of fitted SUDA mass spectra exhibit line widths of $\Delta m < 1 ~\mathrm {u} $. Line widths in excess of 1$~\mathrm {u}$ are a consequence of the selection of the dust analogs and are therefore not representative of ice particle impacts. The mass resolution of an impact mass spectrometer is contingent upon the temperature of the impact plasma. In general, lower impact plasma temperatures result in higher mass resolutions (Mamyrin 1994). The plasma temperature for ice particle impacts is relatively low, on the order of a few$~\mathrm {eV}$, whereas for impacts of metals and minerals, the plasma temperature is considerably higher, ranging between a few$~\mathrm {eV}$ to approximately 50$~\mathrm {eV}$ (Hillier et al. 2006)). As the accelerator tests have been limited to Fe, Al, and olivine, the mass resolution observed in the calibration campaigns are lower than those expected for ice particle impacts in flight. Moreover, the formation of craters with considerable depth resulting from metal impacts affects the distribution of the ion start times at the target. Rather than matching a delta function, the start time distribution of such impacts decays exponentially. The exponential component in the resulting asymmetric line profiles (see Sect. 4.1.4) cannot be reduced by the reflectron’s time-focusing powers. This leads to a systematic reduction in mass resolutions for metal impacts.

#### Quantitative Mass Spectrometry

The accurate interpretation of SUDA mass spectra necessitates the precise quantification of significant species present in complex mixtures of organics, salts, and water. The number of ions of a molecular species in a TOF spectrum is proportional to the area of its mass peak. A simple numerical integration is not a viable option because frequently the tails of adjacent mass lines contribute to the total line area. Instead, the line shapes are fit to Exponentially Modified Gaussians (EMG) (Kalambet et al. 2011), a profile function that aligns with the typically asymmetric appearance of mass lines created by hypervelocity impacts.

**Fig. 19:**
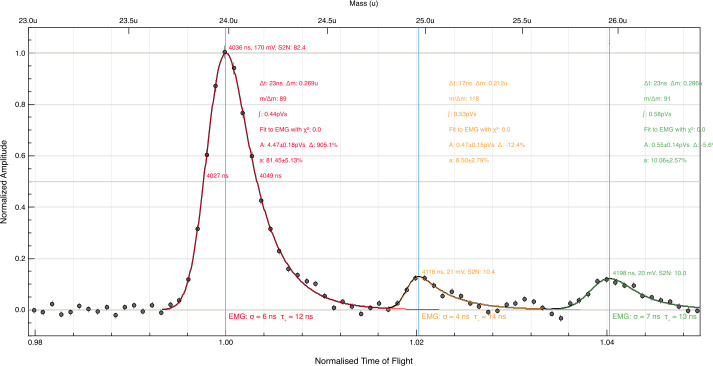
The three magnesium isotopes appearing in a SUDA mass spectrum of a 100$~\mathrm {nm}$ 7$~\mathrm {km\,s^{-1}}$ olivine particle. To compute the abundance of the isotopes, the line shapes have been fitted to an Exponentially Modified Gaussian (EMG) (Kalambet et al. 2011). The resulting isotopic abundances of $(81.4\pm 5.1)$% for $\mathrm {^{24}Mg}$, $(8.2 \pm 2.8)$% for $\mathrm {^{25}Mg}$, and $(10.1 \pm 2.6)$% for $\mathrm {^{26}Mg}$ match the Magnesium reference values $78.99\%$ ($\mathrm {^{24}Mg}$), $10.00\%$ ($\mathrm {^{25}Mg}$), and $11.01\%$ ($\mathrm {^{26}Mg}$) (Kondev et al. 2021) well

To assess the accuracy of SUDA in determining ion counts, we fitted the mass lines in impact spectra of platinum-coated olivine particles (Hillier et al. 2018). The spectra exhibit a pronounced triplet of magnesium isotope lines ($\mathrm {^{24}Mg}$, $\mathrm {^{25}Mg}$, $\mathrm {^{26}Mg}$), which are typically not affected by potential contaminants, thereby providing a reliable basis for comparison. The discrepancy between the relative isotopic abundances derived from the fitted line shapes and the magnesium reference values (Kondev et al. 2021) has been determined to be less than one standard deviation, indicating that SUDA is capable of performing quantitative mass spectroscopy. As a representative example, Fig. 19 depicts the fitted magnesium isotope lines in an olivine impact spectrum recorded with the SUDA FM.

### Velocity Sensor

**Fig. 20:**
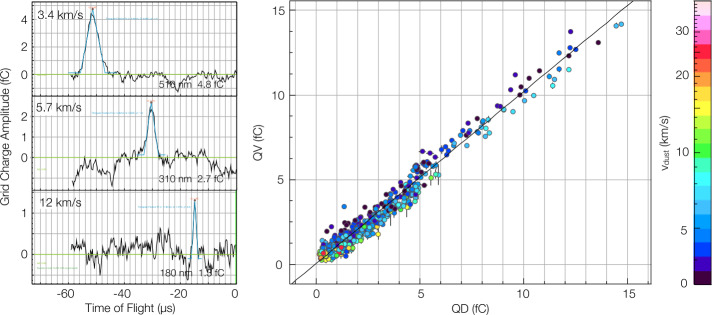
Performance test of the SUDA Velocity Sensor. **Left:** QV waveforms with charge features induced by aluminum particles at $3.4 ~\mathrm {km\,s^{-1}} $, $5.7 ~\mathrm {km\,s^{-1}} $, and $12 ~\mathrm {km\,s^{-1}} $ recorded with the SUDA FM in calibration campaign 3. Blue lines indicate fits of the feature to Eq. (3). **Right:** The dust charge $Q_{D}$ reported by the dust accelerator’s pickup tube detector versus electrostatic dust charge $Q_{d}$ derived from the charge feature induced onto the Velocity Sensor grid

The SUDA Velocity Sensor is designed to derive the speed component parallel to the instrument boresight, $v_{z}$, and the electrostatic charge carried by an incoming dust particle, $Q_{d}$, from the shape of the feature induced onto the sensor’s shielded grid system. As a charged dust particle traverses the stack of three grids, its electrostatic charge induces a corresponding charge onto the central sensor grid, which is attached to a CSA (QV signal). As the particle approaches the sensor grid, the charge amplitude, $Q_{v}$, increases linearly. Conversely, after the particle has traversed the sensor grid, the charge amplitude decreases in a linear manner. The resulting induced triangular feature

3 $$ Q_{v} (t) = Q_{d} \cdot \textstyle\begin{cases} \frac{t v_{z} + d_{2}}{d_{1} - d_{2}} & \text{if} ~ d_{1} \le - v_{d} t \le d_{2} \\ \frac{d_{2} + t v_{z} }{d_{2} - d_{3}} & \text{if} ~ d_{2} \le - v_{d} t \le d_{3} \\ 0 & else \end{cases} $$

is only a function of $v_{z}$ and $Q_{d}$, where $d_{1} = 191 ~\mathrm {mm} $, $d_{2} = 176 ~\mathrm {mm} $, and $d_{3} = 161 ~\mathrm {mm} $ are the distances of outermost shielding grid, the sensor grid, and the innermost shielding grid to the impact target. Both the feature location and the feature width depend on the particle speed, which contributes significantly to the robustness of the feature discrimination from noise spikes. The left plot in Fig. 20 shows $Q_{v}$ features induced by particles at 3.4$~\mathrm {km\,s^{-1}}$, 5.7$~\mathrm {km\,s^{-1}}$, and 12$~\mathrm {km\,s^{-1}}$.

The sensor was calibrated by measuring the response of the velocity sensor to charged iron and aluminum particles (Campaigns 3 and 4). The speed and charge of the dust particles were determined by fitting Eq. (3) to the QV waveform. The time in Eq. (3) is relative to the particle’s time of impact, which is given with$~\mathrm {ns}$ accuracy by the shift parameter $t_{0}$ of the calibrated impact spectrum (see Sect. 4.1.1). The statistical uncertainties of $v_{z}$ were found to be of the order of a few tens of$~\mathrm {m\,s^{-1}}$, which was smaller than the uncertainties associated with the speeds reported by the accelerator’s pickup tube detectors. The velocity sensor detected particles with the highest speeds of approximately 37$~\mathrm {km\,s^{-1}}$.

The QV Sensor’s amplitude to charge conversion factor has been calibrated in relation to the dust charges reported by the dust accelerator’s pickup tube (Fig. 20). The QV Digital Number (DN) to charge conversion factor has been determined to be 134$~\mathrm {DN/fC}$, corresponding to a detection range of $-15.2 ~\mathrm {fC} $ to $15.2 ~\mathrm {fC} $. The limit of charge detection is constrained by the amplifier’s RMS noise, which is approximately 0.25$~\mathrm {fC}$. The Velocity Sensor performance exceeds the relevant SUDA measurement requirement SUDA.015 (see Sect. A) by a significant margin and is approximately ten times more sensitive than the Cassini/CDA QP subsystem (Auer et al. 2002).

### Impact Charge Detection

**Fig. 21:**
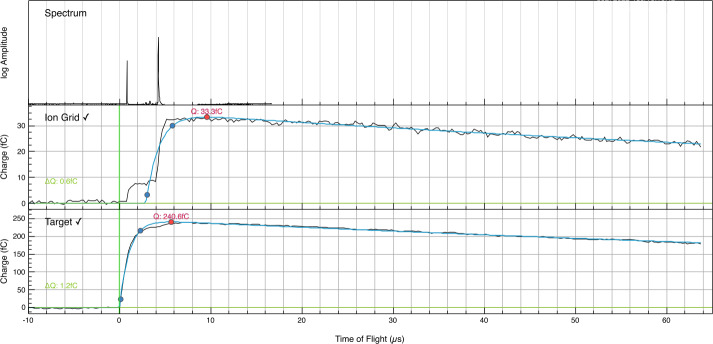
Target (QT, bottom panel) and ion grid (QI, middle panel) waveforms of a 25$~\mathrm {km\,s^{-1}}$ impact of a 52$~\mathrm {nm}$ aluminum particle recorded with the SUDA FM presented together with the TOF waveform (top panel). The time of impact is indicated by the vertical green line. The blue lines are fits of analytic functions Eq. (4) and (5) to the waveforms. The target rise time $t_{t} = 2.2 ~\mathrm {\textrm {\textmu }s} $ is the time elapsed between 10% and 90% (blue bullets) of the QT maximum amplitude $Q_{t} = 241 ~\mathrm {fC} $ (red bullet). The ion grid charge is $Q_{i} = 33.3 ~\mathrm {fC} $. Note the two steps in the QI waveform which correspond to the strong $\mathrm {^{1}H}$ and $\mathrm {^{27}Al}$ mass lines in the TOF waveform

A dust particle striking the SUDA target will produce a combination of particle and target fragments (ejecta), neutral gas, and plasma (for a review of impact ionization detectors see e.g., Auer 2001). The amount of impact plasma, $q_{imp}$, is a function of the impactor’s mass, $m_{d}$, its impact speed, $v_{d}$, and, to a lesser extent, its composition. SUDA derives the characteristics of the impact plasma from the waveforms recorded at the target (QT signal) and the ion grid (QI signal). For illustration, Fig. 21 depicts impact waveforms resulting from a high-speed impact, recorded with the SUDA FM.

The QT waveform (Fig. 21 bottom panel) represents the evolution of the negative ions (cation mode) or positive ions (anion mode) of the impact plasma on the target. It is well reproduced by the analytical function:

4 $$\begin{aligned} Q_{QT}(t) & = Q_{b} + \textstyle\begin{cases} Q_{d} \, e^{-(\frac{t - t_{imp} }{t_{m}})^{2}} & t < t_{imp} \\ Q_{a} \left (1 - e^{-\frac{t - t_{imp} }{t_{1}}} \right ) e^{- \frac{t - t_{imp} }{t_{2}}} & t \ge t_{imp} \end{cases}\displaystyle , \end{aligned}$$

with the target impact charge $Q_{t}$

$$\begin{aligned} Q_{t} & = Q_{a} \frac{t_{2} - t_{1}}{t_{2}} \left ( \frac{t_{1}}{t_{2} - t_{1}} \right )^{t_{1}/t_{2}}. \end{aligned}$$

Here, $Q_{b}$ is the baseline amplitude, while $t_{imp}$ denotes the impact time. The time scales $t_{1}$ and $t_{2}$ are approximately equivalent to the plasma rise time, $t_{t}$ (defined as the interval between 10 and 90% of the waveform’s maximum amplitude), and the CSA’s discharge time of $200 ~\mathrm {\textrm {\textmu }s} $, respectively. The term for $t< t_{imp} $ accounts for the mirror charge induced by charged dust particles after they have crossed the grounded acceleration grid onto the target. The duration $t_{m} = l_{acc}/ v_{d} $ of the mirror charge feature as well as the mirror charge, $Q_{d}$, are measured by the velocity detector (see Sect. 4.2).

The ion grid situated in front of the ion detector (63% transmission) collects 37% of the ion beam which is subsequently recorded as TOF mass spectrum. The peak charge, $Q_{i}$, of the corresponding QI waveform (Fig. 21 middle panel) is proportional to the integral of the TOF waveform. This allows the line integrals to be converted into ion counts. The overall appearance of the QI waveform is analogous to that of the QT waveform. However, due to the fact that the ion molecules arriving as packets, the rise of the waveform is not smooth but instead comprises a sequence of steps (the step amplitude is a direct measure of the ion count corresponding to the respective mass line). Nevertheless, a simplified version of Eq. (4) is able to match the vast majority of QI waveforms with a sufficient degree of accuracy:

5 $$\begin{aligned} Q_{i} (t) = & Q_{b} + \textstyle\begin{cases} 0 & t < t_{imp} \\ Q_{a} \left (1 - e^{-\frac{t - t_{imp} }{t_{1}}} \right ) e^{- \frac{t - t_{imp} }{t_{2}}} & t \ge t_{imp} \end{cases}\displaystyle , \end{aligned}$$

with the ion grid charge $Q_{i}$

$$\begin{aligned} Q_{i} & = Q_{a} \frac{t_{2} - t_{1}}{t_{2}} \left ( \frac{t_{1}}{t_{2} - t_{1}} \right )^{t_{1}/t_{2}}. \end{aligned}$$

Because of the time of flight of the ions to the grid, the resulting QI waveform is delayed by a few$~\mathrm {\textrm {\textmu }s}$ with respect to the QT signal (e.g., flight time of sodium ions is $\approx 3.8 ~\mathrm {\textrm {\textmu }s} $ for $a = 800 ~\mathrm {ns} $). Consequently, the condition that must be met is $t_{imp} ^{QT} \le t_{imp} ^{QI} \le 10 ~\mathrm {\textrm {\textmu }s} $.

We employ the nonlinear Levenberg-Marquardt method (More 1977) to perform a constrained least squares fit of Eqs. (5) and (4) to the recorded QT and QI waveforms. Only waveform fits with a signal-to-noise ratio exceeding 2 are deemed statistically significant. Mirror charge signatures are only considered if they are stronger than 2$~\mathrm {fC}$ and have a signal-to-noise ratio of at least 2. Otherwise, the QT waveform is matched to Eq. (5). From the fits, we derived the impact charge, $Q_{t}$, the target charge rise time, $t_{t}$, and the ion grid charge, $Q_{i}$.

#### Deriving the Dust Mass from the Impact Charge

The impact ionization process is a complex phenomenon that has been the subject of extensive theoretical (e.g., Gault and Heitowit 1963; Drapatz and Michel 1974; Kissel and Krueger 1987; Hornung and Kissel 1994) and experimental (Adams and Smith 1971; Mocker et al. 2013; Collette et al. 2014) investigation. However, despite these efforts, its precise mechanism remains unclear. Theoretical considerations regarding the shock ionization of hypervelocity impactors, in conjunction with experimental evidence (for a review see Auer 2001) indicate that at constant impact speeds, the amount of impact plasma, $Q$, is solely contingent upon the particle mass, $m_{d}$, and the projectile composition. In a first-order approximation, the energy provided by the shock process depends on the specific kinetic energy of the wave propagating through the projectile (Gault and Heitowit 1963). This suggests a roughly linear scaling of $Q$ on $m_{d}$, that is, $Q \sim m_{d} ^{\alpha}$, where $0.5 \lesssim \alpha \lesssim 1$. Indeed, Göller and Grün (1989) observed in calibration experiments with the Galileo dust detector that $\alpha \approx 1$, whereas other studies yielded slightly smaller exponents (e.g., Knabe and Krueger 1982). It can be concluded that a constant speed-independent $\alpha $ indicates that the charge yield $Q/ m_{d} $, i.e., the impact charge per mass, is the observable that is relevant for characterizing the impact process, rather than the impact charge itself.

**Fig. 22:**
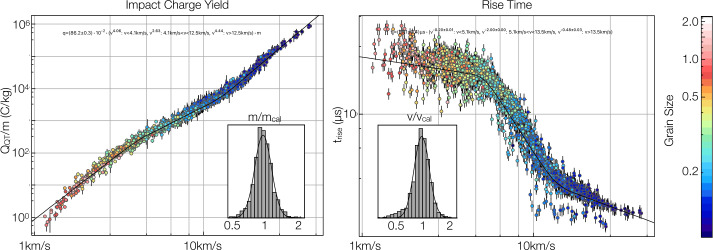
**Left:** Dependence of the impact charge yield, $Q/ m_{d} $, on the impact speed, $v_{d}$, for aluminium particle impacts (Calibration Campaign 3). The inset shows the distribution of the ratios between $m_{d}$ and the mass predicted by the fit of the data to a three-power-laws model. The mass error factor is $\Delta _{m} \sim 0.8$. The color code indicates the impactor size. **Right:** Dependence of the impact plasma rise time, $t_{r}$, of the QT waveform on the impact speed. The inset depicts the distribution of the ratios between $v_{d}$ and the mass predicted by the fit of the data to a three power laws model. The speed error factor is $\Delta _{v} \sim 0.9$

Fig. 22 illustrates the dependence of the charge yield, $Q_{QT}/ m_{d} $, on $v_{d}$ for aluminum particle impacts recorded with the SUDA FM in Calibration Campaign 3 (Table 5). The data has been fitted to a three-power-law model. The curve exhibits a qualitative similarity to data recorded with other impact ionization detectors, including the Galileo instrument (Göller and Grün 1989) and CDA (Srama et al. 2004; Stübig 2002).

As evident from Fig. 22, the impact ionisation can be divided into three distinct regimes: a slow impact regime, a transition regime, and a fast impact regime. The transition regime encompasses speeds between approximately 5 and 10$~\mathrm {km\,s^{-1}}$. At low impact speeds, the energy imparted upon impact is not sufficient to completely vaporize the particle. Instead, the particle fractures into more or less liquid fragments, and most of plasma charge derived from contaminants with the lowest ionization potential that evaporate from the fragment surfaces (Drapatz and Michel 1974). The overwhelming majority of SUDA detections of Europa ejecta are expected to occur within the low-speed regime:

6 $$ \frac{Q}{ m_{d} } = (99.1 \pm 0.5) \cdot 10^{-2} \frac{\mathrm{C}}{ ~\mathrm {kg} } \left ( \frac{ v_{d} }{ ~\mathrm {km\,s^{-1}} } \right )^{3.91} m_{d} ~~~ \text{for}~ v_{d} \le 5 ~\mathrm {km\,s^{-1}} . $$

In contrast, in the high-speed case ($v_{d} \gtrsim 10 ~\mathrm {km\,s^{-1}} $), the particle is completely vaporized, and the impact plasma is predominantly composed of the ionized particle and target material.

Relationships analogous to Eq. (6) have been utilized by a majority of preceding impact ionization detectors to estimate the impactor mass (see the review by Auer 2001). The logarithmic ratios $e_{m} = m_{d} / m_{cal}$ of the actual dust mass relative to the mass estimated by Eq. (6) exhibit a normally distribution, i.e.,

7 $$ N(\ln e_{m}) = \frac{1}{\sqrt{2\pi}\sigma _{m}} \exp \left (- \frac{1}{2} \left [\frac{\ln e_{m}}{\sigma _{m}}\right ]^{2}\right ) $$

(see Fig. 22). This implies that the accuracy of the mass calibration is best described by an error factor $\Delta _{m} = e^{\sigma _{m}}$. In other words, 68% of the mass estimates via Eq. (6) range between $m_{d} / \Delta _{m}$ and $m_{d} \cdot \Delta _{m}$. The error factor of the mass calibration, $\Delta _{m}$, is approximately 0.8. It should be noted that this value represents the mass error factor for impacts for which the impact speed is known. In the absence of this information, the mass error factor is given by the expression $\Delta _{m,v} \le \Delta _{m} \Delta _{v}^{\beta}$, where $\Delta _{v}$ is the error factor of the speed determination.

#### Deriving the Impact Speed from the Impact Charge Evolution

Additionally, empirical evidence indicates that the rise time, $t_{r}$, of the recorded plasma charge signal (defined as the time interval between 10 and 90% of the maximum amplitude) is a characteristic function of the impact speed, $v_{d}$, and is independent of the mass and composition of the striking particle (Auer and Sitte 1968). While the physics underlying this dependence is not yet fully understood, the rise time method has been used successfully employed by a multitude of detectors (e.g., Göller and Grün 1989; Srama et al. 2004). It is probable that the phenomenon originates from ions emitted from the target’s surface as a consequence of an impact-induced surface wave. This is corroborated by the SUDA measurements of the rise time (Fig. 22), which unambiguously exhibits the same phase transitions as evident in the impact charge yield dependence on $v_{d}$. Furthermore, the rise time is a measure of the fraction of the total impact plasma that appears in the spectrum. For impacts with a velocity of 5$~\mathrm {km\,s^{-1}}$ or less, the majority of the plasma ions form a diffuse background. In contrast, for impacts with higher velocities, almost all ions contribute to spectral lines.

In the low velocity range, the dependence of the rise time on the impact velocity is too weak to provide a reliable velocity estimate. At such impact speeds, the Velocity Sensor (see Sect. 4.2) provides superior speed estimates. In the transition and high speed regimes

8 $$\begin{aligned} v_{d} = (35.8 \pm 1) ~\mathrm {km\,s^{-1}} \textstyle\begin{cases} t_{r} ^{-0.5} &\text{for}~ 6 ~\mathrm {km\,s^{-1}} \ge v_{d} \ge 14 ~\mathrm {km} \\ t_{r} ^{-2.1 \pm 0.1} &\text{for}~ v_{d} \ge 14 ~\mathrm {km} , \end{cases}\displaystyle \end{aligned}$$

the rise time method provides useful velocity estimates, particularly for impacts of tiny grains that do not carry sufficient charge to be detected by the Velocity Sensor. The error factor of the speed calibration, $\Delta _{v}$, is approximately 1.2.

## SUDA Science Planning and Operations

### SUDA Operations

#### Data Acquisition

**Fig. 23:**
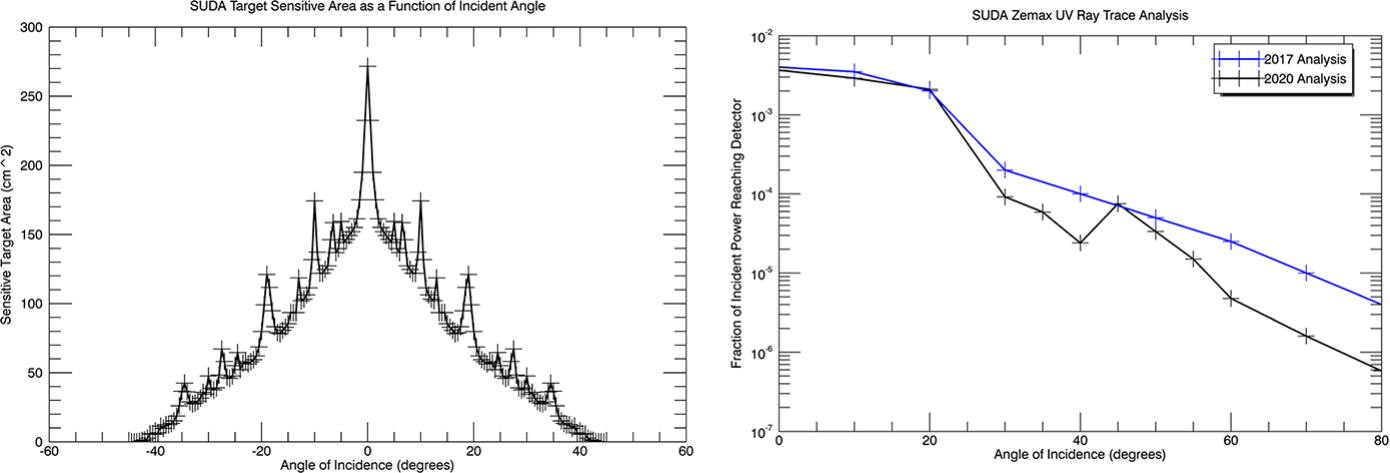
**Left:** The instrument’s sensitive area as function of the dust incidence angle. **Right:** Fraction of the power of a collimated UV beam arriving at the SUDA ion detector as function of the beam incidence angle

Dust particles arriving from directions within SUDA’s angular FOV will either collide with one of the instrument’s grids, the ion detector, or the impact target. Only in the latter instance can an impact mass spectrum recording be initiated. The probability of a target impact, $A_{sens}(\theta )/A_{sens}(0)$, is determined by the instrument’s sensitive area $A_{sens}(\theta )$ (Fig. 23 left). This area depends on the angle, $\theta $, between the instrument boresight and the dust velocity vector in the instrument reference frame. Particles arriving from directions with an angle greater than $42 ^{\circ } $ are unable to impact the target. The peaks in the sensitive area function, $A_{sens}(\theta )$, correspond to incidence angles at which the cells of the grid stack are aligned. Notably, for particles arriving perpendicular to the instrument target, the effective sensitive area is nearly 100% of the geometric area of the target, despite the total grid stack transmission of only 65%. This represents a significant advantage of SUDA over its predecessors.

**Fig. 24:**
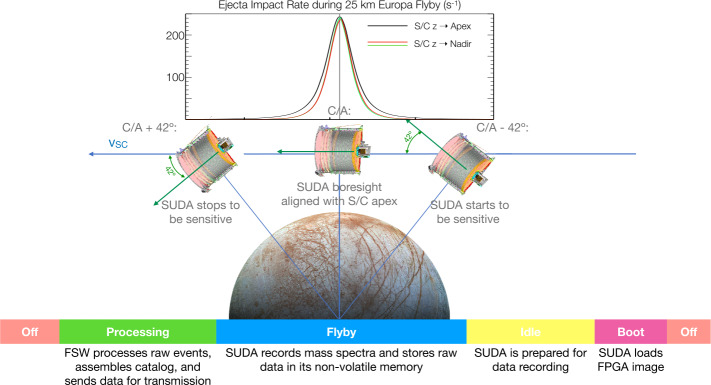
SUDA data acquisition during a Europa flyby with the remote sensing instruments system aligned with Europa nadir (nadir tracking). **Middle:** Instrument orientation during Europa flyby. The ejecta cloud particles arrive from the anti-apex direction of the spacecraft, which is aligned with the SUDA boresight (+x) at the time of closest approach. Throughout the flyby, the spacecraft controls its attitude such that the boresights of the remote sensing instruments (+y) are oriented in the nadir direction. As a consequence, the SUDA sensitive area is time dependent and is maximum at closest approach. **Top:** The impact rate of Europa ejecta depends strongly on the spacecraft altitude and follows a similar trend as the instrument’s sensitive area due to the nadir tracking. Consequently, SUDA Europa measurements remain relatively unaffected by nadir tracking (red line). **Bottom:** Typical SUDA operation sequence during a flyby. SUDA is sensitive to ejecta impacts for typically 5 minutes

During Europa flybys, the boresights of the remote sensing instruments are oriented toward Europa’s nadir. A consequence of the so-called nadir-tracking is that SUDA’s sensitivity to Europa ejecta particle impacts is not constant but rather a function of the angle between the spacecraft-Europa line of sight at closest approach and the spacecraft’s instantaneous position (Fig. 24). However, due to the pronounced dependence of the ejecta cloud density on the altitude ($n_{cloud}(a) \sim a^{-5/2}$, see Krivov et al. 2003), SUDA’s ability to collect ejecta is almost unaffected by the nadir-tracking operation scenario.

#### Health and Safety Risks Due to Ion Detector Exposure to UV, Ambient Plasma, and Dust Impacts

The SUDA instrument geometry differs from that of previous flight instruments in that the detector is oriented to face the entrance aperture. The exposure to ambient plasma, high-energy particles, and UV radiation results in the generation of a background signal. SUDA measures the ion TOF signals in current amplification mode above the background noise. The bias potentials applied to the shielding and reflectron grids will prevent particles, both electrons and ions, with $< 3.2 ~\mathrm {keV} $ from reaching the detector (see Fig. 10). The net contribution from particles with greater energy, considering their detection efficiency, is $3 \cdot 10^{8} ~\mathrm {s^{-1}} $ single particle detections.

SUDA is capable of detecting mass lines with a dynamic range of $10^{3}$ in individual mass spectra, even for the smallest 0.2$~\textrm {\textmu }\mathrm {m}$ particles, with a signal-to-noise larger than 2 relative to the expected random noise background. In the most unfavourable circumstances, SUDA can be operated continuously for 10 months at Europa’s orbital distance before any deterioration in performance is observed. This is typically assumed to occur after the ion sensor has received a total charge in excess of 10 C cm^−2^. It should be noted that the detector has been subjected to rigorous longevity testing, exceeding the aforementioned threshold without exhibiting any gain alterations. Additionally, the detector will occasionally be exposed to direct dust impacts. However, the ion detector will not undergo degradation due to dust impacts, as demonstrated through laboratory measurements (James et al. 2014).

It is imperative to ensure that SUDA measurements are conducted in a manner that prevents direct exposure of the ion detector to solar UV radiation. This is due to the fact that the photoelectron current generated as a result of this exposure has the potential to significantly reduce the operational lifetime of the ion detector. UV photons entering the instrument are scattered at the instrument wall as well as on the grids, which enables photons to reach the ion detector at UV beam incidence angles outside of the instrument’s angular FOV (Fig. 23).

In examining the most intense UV sources for SUDA, it is necessary to consider the Sun, Europa’s sunlit surface, and Jupiter. An analysis of the instrument performance indicates that even in the most unfavorable observation geometry, the UV reflected from Europa’s surface will generate less background noise than energetic particles, due to the limited FOV. The contribution from Jupiter within the field of view is negligible in comparison.

The specific angle of UV exposure that constitutes a risk to the instrument is not as clearly established as the angle for dust particles. To facilitate the Europa Clipper science planning process, there a flight rule has been established that delineates a Solar UV Keep Out Zone (KOZ) of $42 ^{\circ } $. Nevertheless, it is expected that the impact of solar UV on the detector’s operational lifetime will be accounted for as a consumable, by estimating the loss of ion detector lifetime, $\Delta T_{MP}$. Table 10 provides $\Delta T_{MP}$ estimates for all Europa flybys.

#### Instrument Trigger

Dust impacts are stochastic events implying that the instrument must continuously monitor its impact sensitive channels for characteristic signatures to trigger the event data recording. The implemented trigger logic, which is incorporated into the FPGA (see Sect. 3.3.3), is sufficiently flexible to discriminate between impact and noise events even in the most challenging environments, such as Jupiter’s energetic plasma environment. The trigger logic is controlled by the FSW and provides three distinct impact detection modes:

*Threshold Mode*: Event recording commences when the amplitude of a TOF channel surpasses the pre-determined trigger level over a specified number of samples. The FSW sets the trigger level, the minimum requisite sample number, and the TOF channel. This mode is analogous to the mass spectrum trigger mode employed by CDA (Srama et al. 2004).
*Single-Pulse Mode*: Event recording commences when the amplitude of a TOF channel surpasses the pre-established trigger level for a specified number of samples within a designated range. This mode is designed to exclusively trigger on mass lines within a reasonable line width range, thereby addressing potential issues such as drifting baselines.
*Double-Pulse Mode*: Two consecutive *Single-Pulse* triggers are required, separated by a preset time interval. This mode enables the triggering by specific line sequences that are characteristic of materials of interest. For instance, it allows for selective triggering on water ice mass spectra that exhibit the $\mathrm {(H_{2}O)H^{+}}$ and $\mathrm {(H_{2}O)_{2} H^{+}}$ cluster ion lines. This is the most selective mode and is only to be employed if the impact rate exceeds the maximum number of recordable mass spectra of 100$~\mathrm {s^{-1}}$ .

#### Instrument Modes

At any given moment, SUDA will be in one of the following instrument modes:

*Off Mode:* SUDA is off, and only the survival heaters are active.
*Boot Mode:* This mode prepares the instrument for transition into its Flight Software operation state. During this phase, the instrument monitors its health and supports a reduced set of commands primarily for memory operations.
*Idle Mode:* The instrument is in a safe, low-power configuration and is awaiting further instructions.
*Flyby/Survey Modes:* In this operational mode, SUDA is ready for impact detection and the signal channels are monitored for threshold detection. The TOF signal at the ion detector is sampled continuously with either all three (flyby mode) or just one (survey mode) of the high-speed Analog Digital Converters (ADCs). If the commanded trigger condition is fulfilled (Sect. 5.1.3), the raw data of this event will be recorded and stored in the non-volatile instrument memory for subsequent processing .
*Processing Mode:* Raw data collected in either the Flyby or Survey mode are analyzed by the FSW to enable event down selection for transmission to the spacecraft (see Sect. 5.1.5). The processed data remain stored together with the raw data until an event is deleted by an instrument command. It is possible to reprocess stored raw data.
*Safe Mode:* Upon entering this mode, diagnostic data are autonomously transmitted to the spacecraft and the instrument components are set to a safe, low-power state. Any commands that contravene the safe configuration are rejected. SUDA remains in this state until the instrument receives an all-clear signal from the spacecraft.
*Decontamination Mode:* This mode is exercised at least once following each close Europa flyby, or at least once per month, with the objective of removing contaminants that have accumulated on the target. To this end, the target is heated to 110$~{}^{\circ }\mathrm {C}$ for a period of eight hours.

#### Data Products

The SUDA FSW generates two science-related data products: The *Science Data Packets* contain the waveforms of individual impact events, while the *Event Catalog Packets* provide the basic parameters of all recorded impact events during a given observation period. The *Event Catalog* will be generally transmitted to Earth in order to enable the science team to derive quick look data products, such as the impact rate profile and the noise-to-impact-event ratio, for the activity in question. In contrast to the Catalog data, the set of recorded impact events will not necessarily be completely transmitted after the activity. Rather, it may be stored for an extended period in the instrument’s non-volatile memory. Based on the Event Catalog data, the science team can retrieve stored impact events from the instrument at any time for further analysis.

##### Event Catalogs

The Event Catalog is assembled by the FSW during the *Processing* instrument mode (Sect. 5.1.4) and provides characteristic waveform parameters of the events recorded during an SUDA activity, which comprise the so-called *Processed Events* (PE). For the sake of computational efficiency, all FSW computations are performed using integer arithmetic. For each event, the FSW determines the baselines, RMS noise, and maximum amplitudes of the mid-gain TOF, QI, QC, and QV waveforms. In the event that the signal-to-noise ratios (SNR) do not exceed the preset minimum values, the event is flagged as noise and the processing is terminated. In the case of noise events, only a reduced parameter set, designated as *Processed Noise Event* (PNE), will be appended to the catalog (see Table 7). Otherwise, a simple peak finder algorithm (performance ∼N(log N)) is employed by the FSW to identify the amplitudes and times of the first 5 peaks appearing in the mid-gain TOF waveform, with a signal-to-noise exceeding a preset value. Moreover, the rising flanks of QI and QT waveforms are matched to a linear function to derive an approximation for the time of impact as well as for the waveform’s rise time (see Table 7).

**Table 7:** Processed Event entries in the SUDA Catalog

Parameter	Bits	Comment
Processed Event (PE) entry (32 bytes)		
Category	5	
Event Time	16	(sec. & 0x1F) ≪ 12 + subsec. & 0x7FF
Peak Count	4	ceiling at 15 peaks
Peak 1 Amplitude	7	x/4, rel. to baseline
Peak 1 Time	11	(trigger index offset)/4
⋮		
Peak 4 Amplitude	5	Peak 1 Ampl./Peak 4 Ampl.
Peak 4 Time	11	(trigger index offset)/4
Peak 5 Amplitude	5	Peak 1 Ampl./Peak 5 Ampl.
TOF Clipped	1	indicating if waveform is clipped
QV Clipped	1	indicating if waveform is clipped
QT Clipped	1	indicating if waveform is clipped
QI Clipped	1	indicating if waveform is clipped
Peak 5 Time	11	(trigger index offset)/4
TOF Baseline	8	rel. to 50% of full range
TOF Signal to Noise	4	
QV Signal to Noise	4	
QT Signal to Noise	4	
QI Signal to Noise	4	
QV Baseline	8	rel. to 50% of full range
Max. QV Amplitude	8	value/8, rel. to baseline
Time Max. QV	8	(trigger index offset)/8
QT Baseline	8	rel. to 50% of full range
Max. QT Amplitude	8	value/8, rel. to baseline
Time Max. QT	8	(trigger index offset)/8
QT Impact Time	8	trigger index offset
same for QI	32	rel. to 50% of full range
Processed Noise Event (PNE) entry (4 bytes)		
Event Time	16	(sec. & 0x1F) ≪12 + subsec. & 0x7FF
Max. QT Amplitude	6	x/8, rel. to baseline
Max. QI Amplitude	5	x/8, rel. to baseline

Based on the resulting parameter set and a configurable lookup table, the FSW associates the event with one of 40 predefined *categories*, enabling a down selection for further transmission. A comparable methodology was successfully implemented by the CDA flight software (Srama et al. 2004).

**Fig. 25:**
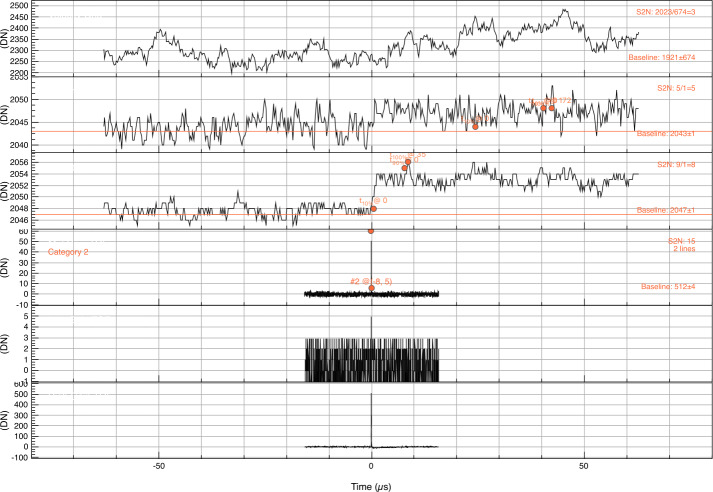
SUDA raw data of an impact event. The parameters of the associated *Processed Event* are marked in red. The shown event is representative for a weak impact of a rather small particle

The maximum of a Catalog per SUDA activity is 216 kB, with 212.7 kB available for event data. This is equivalent to 6645 PEs (28 bytes each) or $53{,}172$ PNEs (4 bytes each). Assuming that 50% of the detections made during a typical SUDA Europa flyby are noise events, the Catalog can accommodate up to 5859 PEs (total $11{,}718$ events), which exceeds the predicted maximum number of detectable ejectas per flyby of approximately 2100 (see Table 10) by a factor of approximately 2.8. In addition to providing information regarding the utilized processing parameters, the Catalog header furnishes the complete instrument time and event number of the initial and final events appearing in the time-ordered event list. This allows derivation of the corresponding instrument times from the reduced times of the event entries. To illustrate the utility of the event parameters defined in Table 7, Fig. 25 depicts the extracted parameter values in the waveform plots of the recorded event.

##### Impact Event Data

SUDA Impact Event Data are comprised of the waveforms and some ancillary data recorded for a single impact event (Table 8). In the *Flyby* mode, the instrument records all three TOF waveforms. This results in a data volume of approximately 31 kB per event. In order to conserve power, only one of the TOF waveforms is be recorded in *Survey* mode (11 kB per event). The data volume per event can be reduced by a factor of three by compressing the waveforms using the FPGA implemented RICE compressor (Sect. 3.3.3), resulting in an effective data volume per event of 14 kB (5 kB in *Survey* mode).

**Table 8:** SUDA Impact Event Data

Mode	Wavef.		Data size	
			Uncompr.	Compr.
Flyby	TOF	3 × (8192 samples, 3.84$~\mathrm {ns}$, 10 bit)	30 kB	13 kB
	QT	512 samples, 246$~\mathrm {ns}$, 12 bit	704 B	300 B
	QI	512 samples, 246$~\mathrm {ns}$, 12 bit	704 B	300 B
	QV	512 samples, 246$~\mathrm {ns}$, 12 bit	704 B	300 B
**Total:** 14 kB				
Surey	TOF	1 × (8192 samples, 3.84$~\mathrm {ns}$, 10 bit)	10 kB	4 kB
	QT	512 samples, 246$~\mathrm {ns}$, 12 bit	704 B	300 B
	QI	512 samples, 246$~\mathrm {ns}$, 12 bit	704 B	300 B
	QV	512 samples, 246$~\mathrm {ns}$, 12 bit	704 B	300 B
**Total:** 5 kB				

### Planned SUDA Measurements

The implementation of SUDA observations is based on the SUDA measurement requirements SUD.002, 003, 016-019, 040, and 041 (see Table 9). In general, SUDA observations are closely linked to the spacecraft trajectory and attitude. Consequently, under the guidance of the *Composition Working Group* (Becker et al. 2024, this collection) and *Geology Working Group* (Daubar et al. 2024, this collection), the spacecraft trajectory has been refined through in a multistep process to enhance the scientific value of SUDA observations.

**Table 9:** Abridged, thematically sorted SUDA Level-2 measurement requirements. The SUDA instrument design requirements as well as the SUDA related mission design are derived from them

ID	Name	Requirement
Allocated to Mission Design		
SUD.002	Number of low altitude flybys over distributed unique geographical locations	The number acquired flybys with closest approach altitudes less than or equal to 35 km shall be greater than or equal to 10, with at least 3 flybys having the closest approach altitude less than or equal to 28 km, and distributed across at least 6 unique panels
SUD.003	Number of combined low and medium altitude flybys over distributed unique geographical locations	The number of acquired flybys with closest approach altitudes less than 110 km shall be greater than or equal to 23, and distributed across at least 7 unique panels.
SUD.018	Unique Geographical Location Observation Coverage	For the unique geographical location composition dataset, the number of covered unique geographical locations (in concurrence with the PSG) shall be greater than or equal to two.
SUD.019	Unique Geographical Location Flyby Altitude	The flyby altitude over a unique geographical location shall be less than or equal to $50 ~\mathrm {km} $.
SUD.016	Flight system velocity	The flight system velocity relative to Europa shall be between 4$~\mathrm {km\,s^{-1}}$ and 7$~\mathrm {km\,s^{-1}}$.
SUD.017	Acquisition geometry	SUDA observations shall be made when the SUDA boresight is less than 45^∘^ of Kepler ram for all flybys with C/A altitudes below 13,500$~\mathrm {km}$, where a successful observation will have no Sun violations when the Kepler ram is within ±20^∘^ with SUDA boresight.
SUD.040	Surface composition and plume particle composition acquisition attitude	The SUDA boresight shall be ≤2.5^∘^ from Kepler Ram at C/A for at least 90% of the planned flybys for each of the in-situ surface composition flybys and plume particle flybys.
SUD.041	Number of globally distributed unique panels traversed at or below 110$~\mathrm {km}$	The ground tracks of flybys at altitudes lower or than equal to 110$~\mathrm {km}$ shall traverse at least 11 unique panels.
SUD.027	Temporal distribution of nanograin particle measurements	The period of time between successive nanograin observations shall be less than or equal to 42 days, with the exception of two intervals between 2 observations which may be up to 126 days apart.
SUD.033	Exogenic ring particles acquisition duration	The total exogenic ring particles observation duration over the mission shall be >720 hours.
SUD.034	Exogenic ring particles boresight pointing	The angle between the SUDA boresight and Kepler Ram shall be ≤45^∘^.
SUD.035	Exogenic nanograin particles acquisition duration	The total observation duration over the mission shall be greater than or equal to 815 hours.
SUD.036	Exogenic nanograin particles acquisition mode	Observational periods shall be greater than or equal to 12 hours with a duty cycle greater than 50% and with interruptions less than or equal to 0.5 hours.
SUD.037	Exogenic nanograin particles boresight pointing	The angle between the SUDA boresight and Io torus disk around Jupiter shall be ≤23^∘^.
Allocated to SUDA		
SUD.004	Spectral mass range	SUDA shall be capable of acquiring mass spectra in the molecular weight range of 5⋅10^−4^ $~\mathrm {u}$ to 500$~\mathrm {u}$.
SUD.005	Mass resolution	The SUDA spectral linewidths for m less than or equal to 200$~\mathrm {u}$ shall be less than or equal to 1$~\mathrm {u}$ (FWHM).
SUD.006	Particle size range	The particle size range detected and analyzed by SUDA shall be between 100$~\mathrm {nm}$ and 2000$~\mathrm {nm}$.
SUD.007	Cation and anion mass spectra	The mass spectra recorded by SUDA shall contain both cations and anions.
SUD.008	Detection sensitivity of amino acids	SUDA shall be able to detect amino acids (such as glycine, aspartic acid, and arginine) in $\mathrm {H_{2}O}$ at greater than or equal to 10 ppm concentration for particles of 1$~\mathrm {\textrm {\textmu }m}$ to 2$~\mathrm {\textrm {\textmu }m}$ radius.
SUD.009	Detection sensitivity of fatty acids	SUDA shall be able to detect fatty acids with between 12 and 30 carbons in $\mathrm {H_{2}O}$ at concentrations greater than or equal to 1 ppm for particles of 1$~\mathrm {\textrm {\textmu }m}$ to 2$~\mathrm {\textrm {\textmu }m}$ radius.
SUD.010	Detection sensitivity of Na and K	SUDA shall be able to detect Na and K in $\mathrm {H_{2}O}$ at concentrations greater than or equal to 10 ppm for particles of 1$~\mathrm {\textrm {\textmu }m}$ to 2$~\mathrm {\textrm {\textmu }m}$ radius.
SUD.011	Detection Uncertainty of Na/K abundance	The uncertainty in the Na/K abundance ratio measured by SUDA shall be less than or equal to 20%.
SUD.012	Detection sensitivity of $\mathrm {SO_{4}^{-}}$	SUDA shall be able to detect $\mathrm {SO_{4}^{-}}$ in $\mathrm {H_{2}O}$ at concentrations greater than or equal to 0.1 ppm for particles of 1$~\mathrm {\textrm {\textmu }m}$ to 2$~\mathrm {\textrm {\textmu }m}$ radius.
SUD.013	Detection sensitivity of $\mathrm {NO_{3}^{-}}$ and Na clusters.	SUDA shall be able to detect $\mathrm {NO_{3}^{-}}$, $\mathrm {H_{2}S^{-}}$, and $\mathrm {Na(Na_{2}CO_{3})_{n}}$ for clusters up to n = 3, and $\mathrm {Na(NaOH))_{n}}$ and $\mathrm {Na(NaCl)_{n}}$ for clusters up to n = 4 in $\mathrm {H_{2}O}$ at concentrations greater than or equal to 1 ppm for particles of 1$~\mathrm {\textrm {\textmu }m}$ to 2$~\mathrm {\textrm {\textmu }m}$ radius.
SUD.014	Confidence of surface composition mapping.	The confidence in detecting the origin on the surface of Europa of the collected material by SUDA shall be greater than or equal to 80% using the reconstructed trajectory knowledge.
SUD.015	Particle velocity measurement	For particles larger than 500$~\mathrm {nm}$ radius with velocities between 3.5$~\mathrm {km\,s^{-1}}$ and 7.5$~\mathrm {km\,s^{-1}}$, SUDA shall measure the boresight component of particle velocity with an uncertainty less than or equal to 50$~\mathrm {m\,s^{-1}}$.
SUD.031	Compositional accuracy	The confidence of particle compositional makeup measured by SUDA shall be ≥90%.
SUD.024	Exogenic particle spectral mass range	The molecular weight range of exogenic particle spectra acquired by SUDA shall be from 1$~\mathrm {u}$ to greater than or equal to 200$~\mathrm {u}$.
SUD.025	Exogenic particle spectral mass resolution	The SUDA spectral linewidths in exogenic particle spectra for m $\le 100 ~\mathrm {u} $ shall be $\le 1 ~\mathrm {u} $ (FWHM).
SUD.026	Exogenic nanograin particle sizes	SUDA shall measure exogenic nanograin particles with a radius greater than or equal to 1$~\mathrm {nm}$.
SUD.028	Exogenic ring particle sizes	SUDA shall measure exogenic ring particles with a radius greater than or equal to 100$~\mathrm {nm}$.
SUD.029	Exogenic particle detection rate	SUDA shall measure exogenic particle impact rates up to 5 particles per second.
SUD.030	Exogenous ring particle size accuracy	The SUDA measurement uncertainty of the size of exogenic ring particles between 100$~\mathrm {nm}$ and 1000$~\mathrm {nm}$ in radius shall be less than or equal to ±20%.

### Europa Flybys

SUDA gathers the data most pertinent to the mission’s success during close flybys of Europa. Given the pronounced dependence of the number of collected ejecta $N_{e}$ as well as of the spatial resolution of the resulting composition map on the flyby attitude, low-altitude flybys (i.e., those lower than 35$~\mathrm {km}$) of particular value for the implementation of the scientific objectives. It is only during low-altitude flybys that the spatial resolution of SUDA composition maps is sufficient to resolve Unique Geographical Locations (UGL) on Europa’s surface. UGLs are geologically young landforms on Europa’s surface, such as Thrace Macula (see Daubar et al. 2024, this collection).

Table 10 provides a summary of the flyby parameters and the predicted sample collections by SUDA for the 49 flybys at Europa during the mission’s reference tour 21F31v6. The numbers of low-altitude flybys ($< 35 ~\mathrm {km} $, 19 flybys) and medium-altitude flybys (35 to 110$~\mathrm {km}$, 23 flybys) flybys exceeds the minimum number required by SUD.002 with sufficient margin. The tour includes four low-altitude flybys over UGLs: two over Thrace and Thera Macula (E14, E51), one over Chaos North (E49), and one over Chaos South (E6), exceeding the required minimum number of UGL (SUD.018) flybys by a factor of two. Furthermore, the reference tour includes promising opportunities for SUDA to gather data from Europa’s leading and trailing hemispheres, with potential for identifying the nature of potential salt deposits (Trumbo et al. 2020).

**Table 10:** Predictions for the Clipper flybys on Europa, using the full model given in Krivov et al. (2003). In total, SUDA will collect about $140{,}000$ samples from Europa’s surface. Given are the time at Closest Approach (C/A) $t_{CA}$, the total number of ejecta $\ge 200 ~\mathrm {nm} $ $N$, the spacecraft (S/C) altitude at C/A $a_{CA}$, the S/C speed relative to Europa at CA $v_{CA}$, the sub-S/C point at C/A $(\lambda , \phi )_{CA}$, the SUDA science target Tar. (LH - Leading Hemisphere, TH - Trailing Hemisphere, CN - Chaos North, CS - Chaos South, NP - North Pole, SP - South Pole, TA - Thrace Macula, TE - Thera Macula), complementary flybys C/F, the loss of SUDA ion detector lifetime due to Solar UV $\Delta T_{MP}$, the total ion detector charge generated by UV $Q_{MP}$, and flight rule violations FR/V (S - Sun Keep Out Zone (KOZ) violation, SE - Sun KOZ egress violation, SI - Sun KOZ ingress violation). Used parameters: $F_{imp}^{\infty } = 7.6 \cdot 10^{-16} ~\mathrm{kg/m^{2}s}$, $K_{e}/K_{i} = 30\%$, $\Psi = 30 ^{\circ } $, $G_{sil} = 0\%$, $\gamma = 2.4$, $\beta = 3.0$, $s_{max} = 100 ~\textrm {\textmu }\mathrm {m} $. Reference trajectory: 21F31v6. The naming convention for the ejecta model parameters follows Spahn et al. (2006)

	$t_{CA}$ UTC	*N*	$a_{CA}$ km	$v_{CA}$ km/s	$(\lambda , \phi )_{CA}$ ^∘^	Tar.	C/F	$\Delta T_{MP}$ 10^−5^%	$Q_{MP}$ *μ*C	FR/V
2031										
04E01	066/02:41:03	90	630	6.5	4.9, 302.6			1.2	11.5	
05E02	086/14:04:09	267	278	6.6	2.0, 240.0			0	0	
08E03	147/13:04:23	2198	50	5.0	−45.2, 333.5	LH		1.7	16.8	
09E04	168/20:07:52	2229	50	4.9	−62.8, 181.7			1.6	15.6	
10E05	190/03:14:33	2146	50	4.8	−31.1, 179.1			2.7	26.7	
11E06	211/10:41:13	3161	35	4.8	−5.1, 179.1	CS	E12	2.2	22.2	
12E07	232/18:09:43	1146	85	4.8	21.7, 178.4	CN	E49	0.9	8.5	
13E08	254/01:38:11	2199	50	4.8	48.5, 177.6		E11, E48	0.2	1.67	
14E09	275/09:08:07	981	97	4.8	77.4, 173.8	NP	E10	0	0.03	
15E10	296/21:46:56	5274	25	4.8	74.3, 181.4	NP	E9	0.2	1.6	
16E11	318/05:18:26	3322	35	4.8	42.2, 178.9		E8	0.7	6.8	
17E12	339/12:57:54	1561	65	4.8	−5.2, 170.6	CS	E6	1.2	11.6	
2032										
19E13	020/16:46:47	2106	50	4.8	2.4, 185.2	CN		0.6	5.8	
20E14	042/00:23:16	3320	35	4.8	−45.7, 177.2	TE, TA	E51	0.1	1.0	
21E15	063/07:51:33	977	100	4.8	−77.1, 174.7	SP	E52	0	0	
22E16	084/15:40:24	209	331	4.6	−19.5, 199.4			0.1	0.5	
23E17	102/07:51:33	4719	100	4.8	−77.1, 174.7	SP	E52	0	0	
24E18	116/23:55:19	1334	75	4.5	−1.2, 155.4		E20, E22	0.2	2.5	
25E19	134/13:36:27	2137	50	4.5	−0.6, 201.4		E21, E23	0	0.03	
26E20	149/02:35:08	1436	100	4.5	0.2, 155.2		E22, E18	0.1	0.5	
27E21	166/16:01:31	3191	35	4.5	0.1, 202.3		E19, E23	0	0	
28E22	184/04:49:23	1332	83	4.5	−0.1, 156.5		E18, E20	0	0.05	
29E23	198/18:33:32	4647	25	4.5	−1.6, 201.9		E19, E21	0	0	
30E24	213/08:08:10	954	100	4.6	15.3, 152.3	CN		0	0	
31E25	231/02:37:25	3240	35	4.7	−12.2, 329.8	LH	E53	0	0	
32E26	245/07:18:14	17	2655	4.5	−5.0, 123.4			0	0	S
2033										
49E27	147/16:03:39	24	89	4.7	−16.8, 25.3	TH	E41	0	0	SE
50E28	165/16:23:29	47	35	4.7	2.0, 329.1	LH	E30^∗^	1.9	18.6	S
51E29	179/10:57:55	4	57	4.8	−0.4, 28.3	TH		3.0	30.1	S
52E30	197/11:49:12	1017	25	4.8	4.71, 328.8	LH	E28	1.6	16.1	S
53E31	211/06:33:11	16	125	4.7	75.9, 7.0	NP		2.5	25.5	S
54E32	225/11:33:48	192	50	4.7	56.6, 359.8		E46	1.8	18.2	S
55E33	239/16:29:40	1581	35	4.7	34.8, 359	LH		1.6	16.0	SI
56E34	253/21:24:42	1904	50	4.7	13.1, 358.3	LH	E44	1.7	16.7	SI
57E35	268/02:19:22	4602	25	4.7	−8.7, 357.8	LH		2.3	23.2	SE
58E36	282/07:13:35	3261	35	4.7	−30.3, 357.2	LH	E42	1.6	16	
59E37	296/12:07:40	2225	50	4.7	−51.8, 356.5			0.3	3.4	SI
60E38	310/16:57:14	3468	35	4.7	−72.1, 352.9	SP	E40	0.1	0.5	
61E39	324/20:46:31	65	889	4.4	−22.0, 327.9			0.2	1.8	
63E40	349/07:31:44	5245	25	4.5	−72.7, 8.6	SP	E38	0.4	4.1	
2034										
65E41	009/04:15:55	2145	50	4.4	−14.4, 16.1	TH	E27	1.2	12.4	
66E42	023/08:45:35	946	100	4.4	−29.3, 359.0		E36	0.2	2.1	
67E43	037/13:27:22	2203	50	4.4	−36.9, 337.8			0	0.2	
69E44	062/10:06:17	3204	35	4.5	16.9, 358.3		E34	0	0.4	
71E45	087/06:37:17	5227	25	4.4	63.9, 34.7			0	0	
72E46	101/11:18:21	2106	25	4.4	63.2, 356		E32^∗^	0	0	
73E47	115/15:57:02	857	50	4.4	85.4, 192.1	NP		0	0	
74E48	129/20:54:29	1325	35	4.4	52.3, 179.7		E8	0	0	
75E49	144/01:46:06	1247	35	4.4	19.2, 179	CN	E7, E17	0	0	
76E50	158/06:42:05	746	50	4.4	−12.5, 178.5	CS		0	0	
77E51	172/11:38:46	1320	35	4.4	−45.2, 177.6	TE, TA	E14	0	0.06	
78E52	186/16:35:17	507	75	4.4	−89.3, 170.3	SP	E15	0	0	
79E53	201/03:38:52	572	70	4.4	−8.2, 330.2	LH	E25	0	0.06	

#### Flybys at Ganymede and Callisto

SUDA will endeavor to gather compositional data from Ganymede as part of the instrument calibration campaigns during close flybys on Ganymede during the initial stages of the tour (Table 11). There are three Callisto flybys in the reference tour that are sufficiently close to enable SUDA to collect surface ejecta (Table 12). Potential observations will be conducted on a best effort basis.

#### Galilean Ring

The SUDA ring particle campaigns (compliant with SUD.033) have not been implemented yet.

#### Io Nanograin Flux

Specific campaigns (in compliance with SUD.027, 035-037) conducted outside a one-day time window around Europa flybys for the purpose of measuring the nanograins flux emerging from Io. During each campaign, SUDA will be oriented toward the Jupiter line-of-sight direction for approximately 10 hours to capture the flux fluctuations caused by the changing planetary magnetic field over the course of one Jupiter rotation (Horányi et al. 1993). The flux may vary by more than two orders of magnitude as a consequence of the charged nanodust being scattered by the wobbling magnetic field of Jupiter. By characterizing the Io nanodust flux at various time scales, the SUDA dataset will contribute to constrain the deposition flux of Io nanodust onto Europa, Ganymede, and Callisto, as well as serve as a monitor for the volcanic activity over the entire mission (Krüger et al. 2003a).

## Appendix A: SUDA Measurement Requirements

The SUDA Level-2 measurement requirements have been defined by the Europa Clipper project together with the SUDA team to implement the SUDA Level-2 science requirements given in Table 1. The SUDA instrument design parameters as well as the SUDA related mission design are derived from them.

## Appendix B: SUDA Tour Measurements

### B.2 Ganymede Flybys

**Table 11:** Predictions for the Clipper flybys on Ganymede, based on the model given in Krivov et al. (2003). In total, SUDA will collect more than 200 samples from Ganymede’s surface. Given are the time at closest approach (C/A) $t_{CA}$, the total number of ejecta $\ge 200 ~\mathrm {nm} $ $N$, the spacecraft (S/C) altitude at C/A $a_{CA}$, the S/C speed relative to Ganymede at CA $v_{CA}$. SUDA will not perform measurement during G07 flyby because of the Sun Keep-out Zone violation. Reference trajectory: 21F31v6

	$t_{CA}$ UTC	*N*	$a_{CA}$ km	$v_{CA}$ km/s
2030				
01G01	303/14:09:28	179	250	8.72
2031				
02G02	002/23:49:28	42	883	8.72
03G03	038/18:17:35	6	4038	8.60
03G04	103/08:16:21	10	2603	7.93
07G05	121/04:45:41	9	3015	7.92
2032				
36G06	302/11:07:04	18	1643	5.69
2033				
46G07	094/04:09:58	0	972	4.84

### B.3 Callisto Flybys

**Table 12:** Predictions for the Clipper flybys on Callisto, based on the model given in Krivov et al. (2003). In total, SUDA will collect about 100 samples from Callisto’s surface. Given are the time at closest approach (C/A) $t_{CA}$, the total number of ejecta $\ge 200 ~\mathrm {nm} $ $N$, the spacecraft (S/C) altitude at C/A $a_{CA}$, the S/C speed relative to Callisto at CA $v_{CA}$. SUDA will not perform measurement during later Callisto flybys (C05 to C09) because of the Sun Keep-out Zone violation. Reference trajectory: 21F31v6

	$t_{CA}$ UTC	*N*	$a_{CA}$ km	$v_{CA}$ km/s
2032				
34C01	273/15:19:05	12	1311	5.16
37C02	311/19:38:20	87	222	4.26
38C03	328/12:07:02	2	4654	3.88
39C04	354/08:57:11	29	607	4.19
2033				
41C05	009/12:37:51	0	1463	4.07
44C07	069/09:02:20	0	3072	3.93
47C08	119/05:42:34	0	634	5.54
48C09	135/22:12:38	0	3696	5.35
